# Stacked multi-fusion CNN: an adaptive attention model for privacy preserving deepfake forensics

**DOI:** 10.1038/s41598-026-55325-y

**Published:** 2026-05-29

**Authors:** Jayanti Rout, Minati Mishra, Ram Chandra Barik, Devendra Kumar Yadav, Dilip K. Prasad, Arif Ahmed Sekh

**Affiliations:** 1 P. G. Department of Computer Science, Fakir Mohan University, Balasore, 756019 Odisha India; 2 Department of CSE, C. V. Raman Global University, Bhubaneswar, 752054 Odisha India; 3 School of Computer Science and Engineering, XIM University, Bhubaneswar, 752050 Odisha India; 4 Department of Computer Science, UiT The Arctic University of Norway, Tromsø, 9037 Norway

**Keywords:** Deepfake detection, Convolutional neural network, Squeeze-and-excitation attention, Engineering, Mathematics and computing

## Abstract

The emergence of Generative Artificial Intelligence (Gen-AI) and Generative Adversarial Network (GAN)-based deepfakes poses significant security risks in sociocultural and sociopolitical domains. This necessitates the development of advanced and effective detection methods to prevent vulnerability in social networks. Classical Machine Learning (ML) algorithms have their limitations, especially in classifying the deepfakes. To address these issues, this paper suggests a privacy-preserving Stacked Multi-Fusion (SMF) Convolutional Neural Network (CNN) approach to classify deepfakes. An improved CNN model is proposed, integrating an adaptive multi-scale attention framework with enhanced residual blocks and a Squeeze-and-Excitation (SE) mechanism. The selection of these components is backed with an ablation study to report the individual contribution to the overall optimal architecture. A hybrid lossless multilayer cryptosystem based on a chaos-based approach, Deoxyribonucleic Acid (DNA)-based computing, etc., is developed to secure images in cloud storage. The efficacy of the proposed SMF model is validated using the 140K Real and Fake Faces (RFF) image dataset. The proposed approach was found to achieve stable performance with minimal variation in various tests. It achieved a test accuracy and ROC-AUC of 97.80 and 99.79, respectively. This paper provides comprehensive relevant factors for building effective synthetic media detection systems.

## Introduction

In recent years, Artificial Intelligence (AI) has undergone groundbreaking transformations and has evolved from a theoretical concept to practically everywhere. From voice assistants to automating the entire supply chain, it has gained unprecedented integration into our lives. This expansion in various domains, including generative AI and explainable AI, has also presented various serious consequences to society. One of the most controversial manifestations of AI is the creation of deepfakes. It refers to the manipulation of the media that can make it appear that someone said or did something they never did^1^. Through Generative Adversarial Networks (GAN), these realistic fakes are increasingly difficult to detect with the naked eye^2,3^. Their amalgamation with other AI-generated images and real images raises concerns about trust^4^. This highlights the need for a fast and effective detection approach.

Traditionally, Deepfake Face Detection (DFD) relied on manual inspection. Forensics experts would examine videos or images for inconsistencies in the media. Inspecting unusual patterns in the pixels, glitches, inconsistent facial expressions, etc., was one of the most common techniques of DFD. In addition, traditional Computer Vision (CV) approaches have proven inadequate for detecting the increasingly refined images of GANs^5^. However, these approaches were often time-consuming and difficult to detect with the naked eye. Although Machine Learning (ML) was initially applied for DFD, the reliability of human-crafted features and the lack of robustness made it unsuitable for practical use. As a result, Deep Learning (DL) models came into play.

DL techniques, including Convolutional Neural Network (CNN) and Recurrent Neural Network (RNN), have emerged as an effective solution for DFD. Its automated feature extraction, ability to process temporal and spatial relations, etc., made it adaptable and suitable for today’s dynamics. However, they can be resource-intensive when trained with large data to achieve better generalization. In addition, the dependence on a single architecture can bottleneck the performance. Strategic integration of sound technologies could be an effective way to prevent it. Although many studies have explored different DL models for DFD, it is inadequately explained which elements (e.g., normalization, attention, feature fusion) actually improve detection accuracy^6,7^. This makes it harder to build ideal systems and to understand why some methods work better than others. Existing approaches lack a comprehensive development for DFD, designed with real-world scenarios in focus. Secure storage of images is an essential part of cloud-based storage methods. These storage methods are widely used to maintain a self-learning repository for models. Traditional algorithms such as Advanced Encryption Standard (AES)-256 and its other variants are not suitable for this purpose. Although public key cryptography provides improved security compared to the latter, it requires more computational resources and time^8,9^. In addition, using a single layer of protection is vulnerable to data leakage for a successful cryptanalysis. This oversight is problematic in practical deployment scenarios where computational resources may be constrained.

To address these limitations, a comprehensive development strategy for a privacy-preserving DFD approach is proposed. It presents a sustainable and reliable use of various components to create an end-to-end life-cycle. A Stacked Multi-Fusion (SMF) model for DFD is developed to classify the images. It uses various architectural components, supported by an incremental ablation study. These components are selected and integrated to gain the best performance and solve the limitations of a single architecture. This study evaluates the contribution of different architectural components for DFD. The efficient analysis of the integration of each component helps to detect the contribution of individual components. CNN is selected as the baseline model due to its adaptive and combinable architectural design. The 140K Real and Fake Faces (RFF) dataset is used to evaluate each model^10^. Various classification and efficiency metrics are used to quantify performance. Another important aspect of the study focuses on the security of images stored in cloud-based methods. A lossless multilayer hybrid image cryptosystem is developed to secure the images. It involves the combination of chaos theory, fractal permutation, Deoxyribonucleic Acid (DNA)-based encoding, etc., to secure images without relying solely on the security design of the cloud itself^11^. The classification and privacy-preserving approach to maintain privacy along with a complete pipeline is not just an amalgamation of different technologies but an innovative approach to deal with DFD effectively. Various tests and quantitative metrics demonstrate the effectiveness of the approach.

The primary contributions of this work are as follows:

- Comprehensive DFD design: An end-to-end life-cycle for a privacy-preserving DFD framework is proposed.
- Insights on architectural evolution: Various observations are identified during the experiments to design an ideal DFD model.
- Extensive robustness analysis: A comprehensive analysis of SMF has been carried out using various parameters and architectural properties.
- Multilayer hybrid cryptosystem: A lossless multilayer hybrid image cryptosystem is introduced to ensure secure storage of images in cloud-based environments.

The subsequent sections of this paper are categorized as follows: Section “Related works” provides a brief overview of current advancements in DFD. Section “Methodology” explains the methodology of the proposed approach. Section “Experimental details and results analysis” presents comprehensive experimental results and performance analysis. Section “Conclusion and future work” concludes the work and outlines future research directions.

## Related work

Digital image forgery is one of the most prevalent forms of digital media manipulation. From simple copy-move or splicing operations to advanced deepfakes generated by AI, it has various forms^12^. Early traditional methods rely on carefully designed signal processing and statistical features to reveal inconsistencies introduced by editing. For example, copy–move forgery detection techniques compare overlapping blocks or keypoint descriptors such as Scale-Invariant Feature Transform (SIFT)^13^ and Speeded-Up Robust Features (SURF)^14^ to find duplicated regions. On the other hand, splicing detection inspects abrupt changes in texture, lighting, and edge continuity^15^. Compression artifact analysis methods, including Error Level Analysis (ELA)^16^ and JPEG block analysis, exploit differences in recompression signatures to locate tampered areas^17^. Although these approaches are computationally efficient and interpretable, they often fail when faced with high-quality manipulations that use post-processing steps such as recompression, smoothing, or geometric transformations^18^.

**Table 1:** Literature review summary of Image forgery detection techniques.

Refs.	Method	Focus	Dataset	Remarks
^19^	CNN-CenSurE Fusion Approach	Copy-Move Forgery	7 Public Datasets (e.g., CMFD, GRIP, CoMoFoD, MICC-F600, MICC-F220, COVERAGE, and CASIA-II.)	Robust against various copy-move types including post-processing and geometric attacks; fast and reliable for textured images
^20^	FAMSeC (CLIP:ViT + LoRA + Contrastive Learning)	AI-Generated Image	ForenSynths (ProGAN, StyleGAN, etc.), UniversalFakeDetect (Diffusion), GenImage (Diffusion)	Strong generalization from few-shot learning; effective in diverse unseen generative models
^21^	Inception + Xception + CCA + CNN (Federated Learning)	AI-Generated Image & Video	FaceForensics++, Deepforensics-1.0, WildDeepfake	Combines federated learning and feature fusion for privacy-aware forgery detection.
^22^	Guardian AI (ViT + LSTM + Attention Ensemble)	Deepfake Detection (Image & Video)	CDDB (Custom Deepfake Detection Benchmark)	Uses ViTs, LSTM, and multi-head attention for spatiotemporal anomaly detection; tested on realistic deepfake scenarios; robust and precise ensemble model.
^23^	Passive KB-CMFD using DHE, LIOP, sK-means++	Copy-Move Forgery	CoMoFoD, MICC-F220, synthetic defecto MSCOCO	Efficient for small tampered regions; uses dynamic histogram equalization and local intensity order patterns with enhanced clustering.
^24^	Dual-branch network with frequency adaptive enhancement & feature selective fusion	Robust Image Tampering	CASIA v1, CASIA v2 (train/val); NIST16, Columbia, COVERAGE, IMD2020, Openforensics, CocoGlide (test)	Effective for within- and cross-dataset validation; uses frequency-aware and attention modules;
^25^	SO-detection method with FFSC dataset	Semantic-aware Face Forgery	FFSC (newly constructed)	New semantic-based definition and dataset; highlights limitations; suggests multimodal and generative model extensions.
^26^	Semantic Token Clustering + Scoring Net	Facial Semantic Deepfake	WildDeepfake, CelebDF-v2, FF++, DFDC	Strong generalization via semantic clustering; limited by token processing speed; future work on clustering.
^27^	SFFI + AAML	Deepfake Detection (Frame-Level)	FF++, CelebDF-v1/v2, DFDC Preview	Integrates spatial-frequency features; uses AAML to solve class imbalance; outperforms 8 SOTA methods. Future: video-level extension.
^28^	SNIS	Post-Processed Forgery	NIST16, Columbia, CASIA	Uses signal-noise separation to localize tampering; robust to post-processing; improves multi-scale feature learning using atrous convolutions.

To overcome the limitations of hand-made features, ML-based methods adopt a data-driven paradigm. In these techniques, characteristics such as Local Binary Patterns (LBP)^29,30^, Histograms of Oriented Gradients (HOG)^31^, or Discrete Cosine Transform (DCT)^32,33^ coefficients are extracted and used to train classifiers (e.g., Support Vector Machines (SVM), k-Nearest Neighbors (k-NN), or Random Forests (RF)). These models can generalize more effectively for different types of forgery and image qualities. However, their performance is limited to the discriminative power of the features engineered manually and the availability of labeled training data^34^. The researchers in^35^ proposed an ensemble approach to detect resampling forgeries using Markov features.

The recent use of DL-based approaches has transformed image forgery detection by enabling end-to-end learning of hierarchical features directly from raw pixels. CNNs such as Visual Geometry Group (VGG)^36^, Residual Network (ResNet)^37–40^, Extreme Inception (Xception)^41,42^, and EfficientNet^43,44^ automatically discover subtle texture and semantic cues indicative of manipulation. The researchers in^45,46^ proposed an ensemble approach to detect recompression-based forgeries using various Pre-Trained Models (PTM). In addition, Table 1 presents an overview of various DL models and methodologies used in various studies.

## Methodology

### Overview of the proposed research framework

**Fig. 1:**
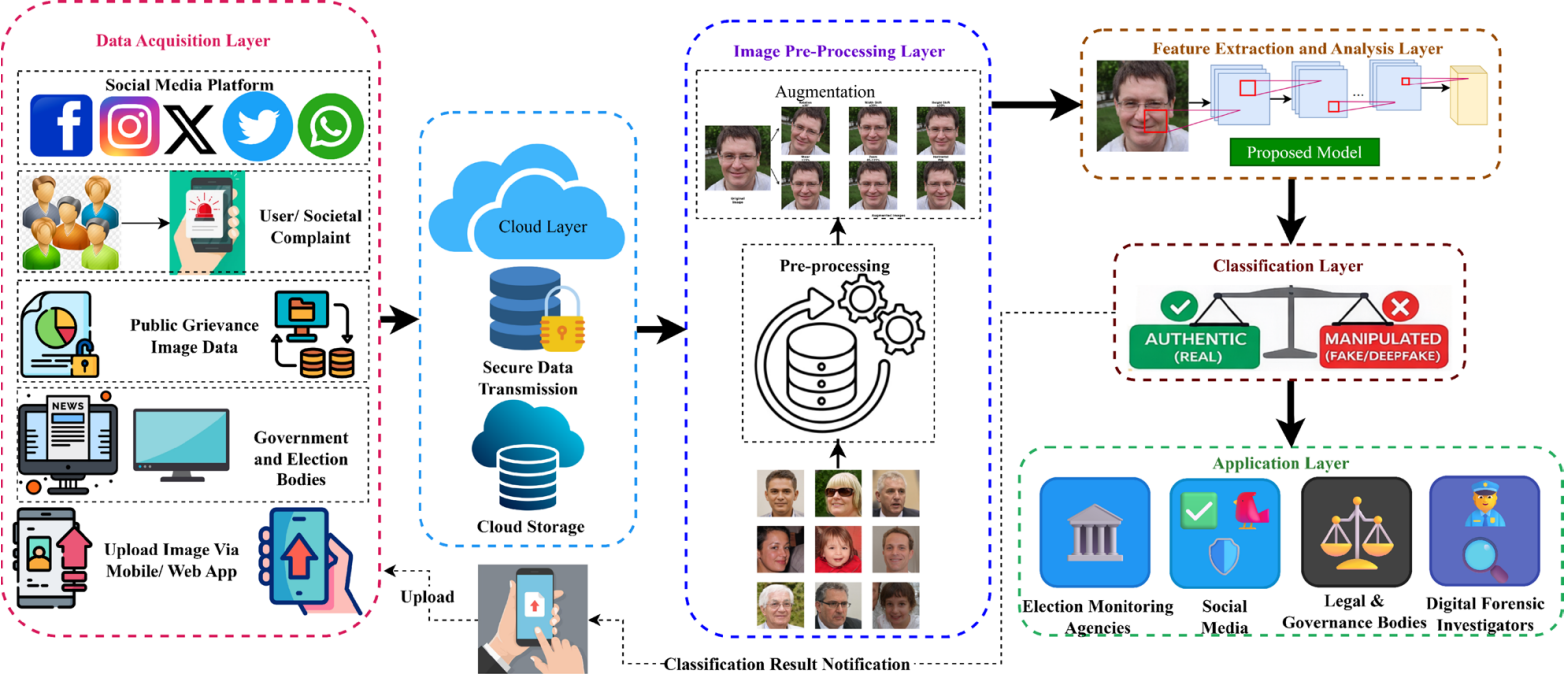
General framework of the proposed SMF model for privacy-preserving deepfake forensics.

The proposed Stacked Multi-Fusion (SMF) approach is designed in multiple stages to complete a life-cycle of an image. It is depicted in Fig. 1. The proposed methodology focuses on the detection of deepfakes while maintaining the privacy of the image. It begins with a data acquisition layer, where images are collected from multiple sources through a mobile or web application. These images may also include user complaints, social networks, government bodies, etc. Once the data is collected, it is securely transmitted to the cloud-based storage technologies. It provides scalability and ease of access for further processing. Ensuring the privacy and integrity of the image for all states of the data is one of the primary targets of the approach. This layer also prepares the images for reliable analysis while maintaining privacy measures. The use of platform-agnostic design principles, as suggested in^47,48^, can increase the adoption of the design principle.

The images are sent for analysis once the images are securely stored. Its accurate classification is another primary focus of the approach. It uses various steps to accurately identify the image as a deepfake or not. Different preprocessing techniques are applied in a privacy-aware manner to ensure that no additional metadata is exposed or stored. In addition, different data augmentation strategies are used to increase the diversity of the dataset and the robustness of the model. It helps the system handle real-world variations in image quality and patterns. The features are extracted and analyzed from the image for a reliable classification. The final decision is binary in nature. Provides legal bodies and other statutory entities with the ability to make informed and sound decisions.

Based on this end-to-end methodology, the proposed work revolves around two main aspects:

1. Deepfake image classification model
2. The privacy-preserving approach to secure images in cloud-based environments

These components ensure accurate detection as well as protection of sensitive information in the image.

### Deepfake image classification model

In this study, an SMF model has been proposed for a robust DFD. Its design methodology follows a staged architecture development approach. Different architectural components are evaluated to understand their individual and combined contributions.

#### Dataset description and preprocessing

**Fig. 2:**
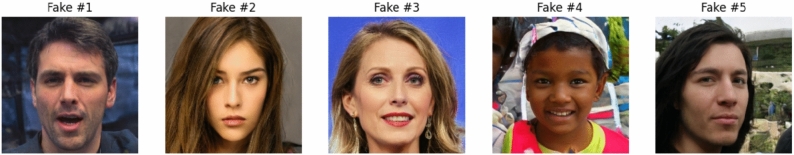
Samples of synthetic faces of the 140k RFF dataset.

*Dataset description* In this study, the 140k RFF dataset^10^ has been used to train and evaluate the model. It is a collection of 140,000 high-resolution facial images. The images are a collection of diverse demographic representations and use various deepfake generation techniques. It provides a balance between authentic and synthetic facial samples for robust model training and evaluation capabilities. As presented in Table 2, a balanced scenario is used during training and evaluation. The training set consists of 100,000 images. Both the validation and testing sets consist of 20,000 images each. All images have been standardized to *256× 256* pixel resolution to maintain consistency in the dataset. Samples of synthetic faces are illustrated in Fig. 2.

**Table 2:** Distribution of samples in the 140k RFF dataset.

Split	Real faces	Fake faces	Total	Percentage
Training	50,000	50,000	100,000	71.4%
Validation	10,000	10,000	20,000	14.3%
Testing	10,000	10,000	20,000	14.3%
Total	70,000	70,000	140,000	100%

*Data preprocessing and augmentation* Data preprocessing is used to ensure the consistency and optimal performance of the model. All input images undergo standardized preprocessing using Eq. (1).

1 $$\begin{aligned} I_{normalized} = \frac{I_{input}}{255.0} \end{aligned}$$

where $I_{input}$ represents the raw pixel values and $I_{normalized}$ denotes the normalized input tensor with values in the range [0, 1].

Augmentation creates variations of the training images. It makes the model learn features rather than memorizing specific patterns. The validation and test sets are not augmented to provide an unbiased evaluation. A comprehensive data augmentation pipeline was designed to improve the generalization and robustness of the proposed model. It consisted of the following:

- Geometric transformations: It includes horizontal flip with 50% probability and rotation within $\pm 10^{\circ }$.
- Spatial augmentations: It includes width and height shifts with variations in the zoom of $\pm 10\%$.
- Photometric variations: It includes adjustments in brightness within the range [0.9, 1.1].

#### Proposed model

**Fig. 3:**
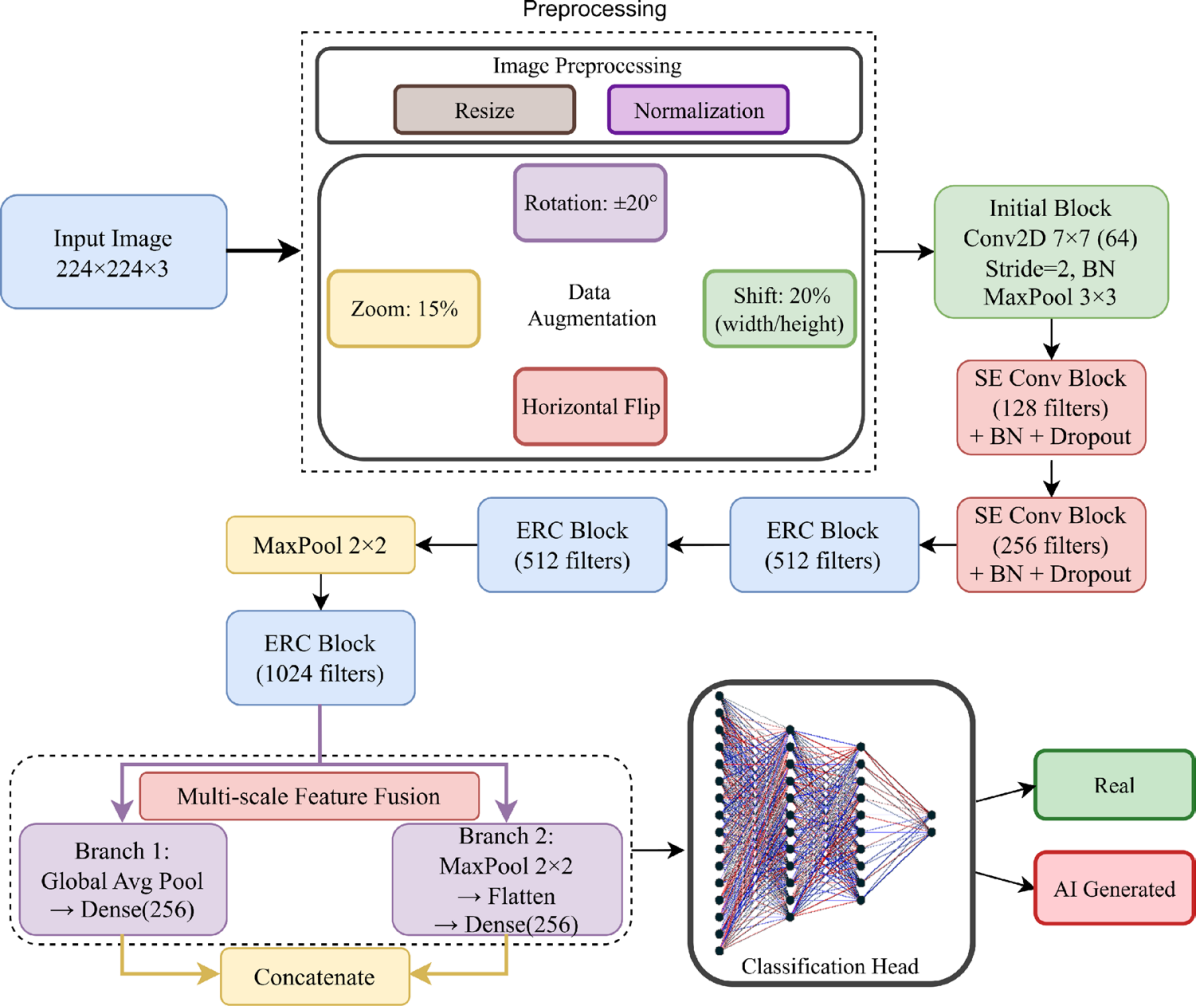
Proposed stacked multi-fusion (SMF) model.

The proposed SMF approach uses a modified deep CNN model for robust DFD. It consists of different components specialized for this task. It integrates an attention mechanism, an Enhanced Residual Connection (ERC), multi-scale feature fusion, Fully Connected (FC) layers, etc., to capture both low-level artifacts and high-level semantic patterns of the synthetic media. The high-level architectural view of the model is presented in Fig. 3.

An input RGB image of size $224 \times 224 \times 3$ is provided to a sequence of layers. These layers focus on feature extraction, attention modeling, and classification. An initial convolutional block that consists of a *7 × 7* convolutional layer with 64 filters and a stride of 2 processes the image. Then, Batch Normalization (BN) and a *3 × 3* max pooling operation are performed on the image. It reduces spatial dimensions and extracts fundamental features, such as edges, textures, etc. A larger kernel is used at this depth to capture broad contextual information. It is used to detect fine manipulation artifacts that could lie in multiple pixels. The combined effect of strided convolution and pooling reduces the resolution from *224 × 224* to *37 × 37*.

The extracted features are then passed through two Squeeze-and-Excitation Convolutional (SE Conv) blocks with 128 and 256 filters, respectively. Each SE Conv block contains two *3 × 3* convolutional layers with Rectified Linear Unit (ReLU) activation and BN, followed by an SE attention module. The SE module first applies Global Average Pooling (GAP) to generate channel-wise descriptors, which are then passed through two FC layers with a reduction ratio of 16 and a sigmoid activation to produce adaptive channel weights. These weights recalibrate the feature maps through element-wise multiplication, allowing the network to emphasize informative channels. Max pooling is used for spatial reduction, and dropout rates of 0.1 and 0.2 are applied after the first and second SE Conv blocks, respectively, to reduce overfitting.

The core of the architecture consists of three ERCs blocks that operate at increasing feature depths. Each ERC block follows a residual learning strategy with two *3 × 3* convolutional layers and BN. In addition, a parallel *1 × 1* convolution is also used in the shortcut path to match the dimensions. An SE attention module is applied to the residual branch before feature addition. It provides channel-wise refinement of residual features. The first two ERC blocks maintain a spatial resolution of *9 × 9* with 512 channels. On the other hand, the third expands the depth of the feature to 1024 channels and applies the *2 × 2* max pooling. This stage results in the majority of the model parameters since it is responsible for feature learning.

To combine global context with local spatial details, a dual-branch multi-scale feature fusion mechanism is used. One branch applies GAP to the $4 \times 4 \times 1024$ feature maps. It provides the resulting global descriptor in a 256-dimensional space. The second branch preserves local information by max pooling and flattening the features. It maps them to another 256-dimensional vector. These two representations are combined and normalized using BN. A dropout layer with a rate of 50% is used for regularization.

Table 3 presents the parameters of the proposed SMF model in detail. The fused features are then passed to the classification head. It consists of two FC layers with 512 and 128 units, respectively. They are followed by BN and dropout. The final output layer uses softmax activation with two neurons for binary classification.

**Table 3:** Architectural configuration of the proposed SMF model.

Layer type	Configuration	Output Shape	Parameters
Input	224 *×*224*×* *×*3 RGB	(None, 224, 224, 3)	0
Initial Block	Conv2D(64, 7*×*7, s=2) + BN + MaxPool	(None, 56, 56, 64)	9,728
SE-Conv Blocks	SE-Conv(128) + BN + Pool + Dropout	(None, 28, 28, 128)	225,536
	SE-Conv(256) + BN + Pool + Dropout	(None, 14, 14, 256)	900,560
Enhanced Residual Blocks	ERC(512) + SE Block 1	(None, 14, 14, 512)	3,573,504
	ERC(512) + SE Block 2	(None, 14, 14, 512)	4,741,248
	ERC(1024) + SE + Pool	(None, 7, 7, 1024)	15,826,432
Multi-Scale Fusion	Branch 1: GAP *→* Dense(256)	(None, 256)	262,400
	Branch 2: Pool *→* Flatten *→* Dense(256)	(None, 256)	2,359,552
Concatenation	Feature Fusion	(None, 512)	0
Classification	Dense(512) + BN + Dropout	(None, 512)	262,656
	Dense(128) + Dropout	(None, 128)	65,664
	Dense(2) + Softmax	(None, 2)	258
**Total Parameters**			**27,645,082**
Trainable Parameters			27,629,082
Non-Trainable Parameters			16,000

### Privacy-preserving approach to secure images

**Fig. 4:**
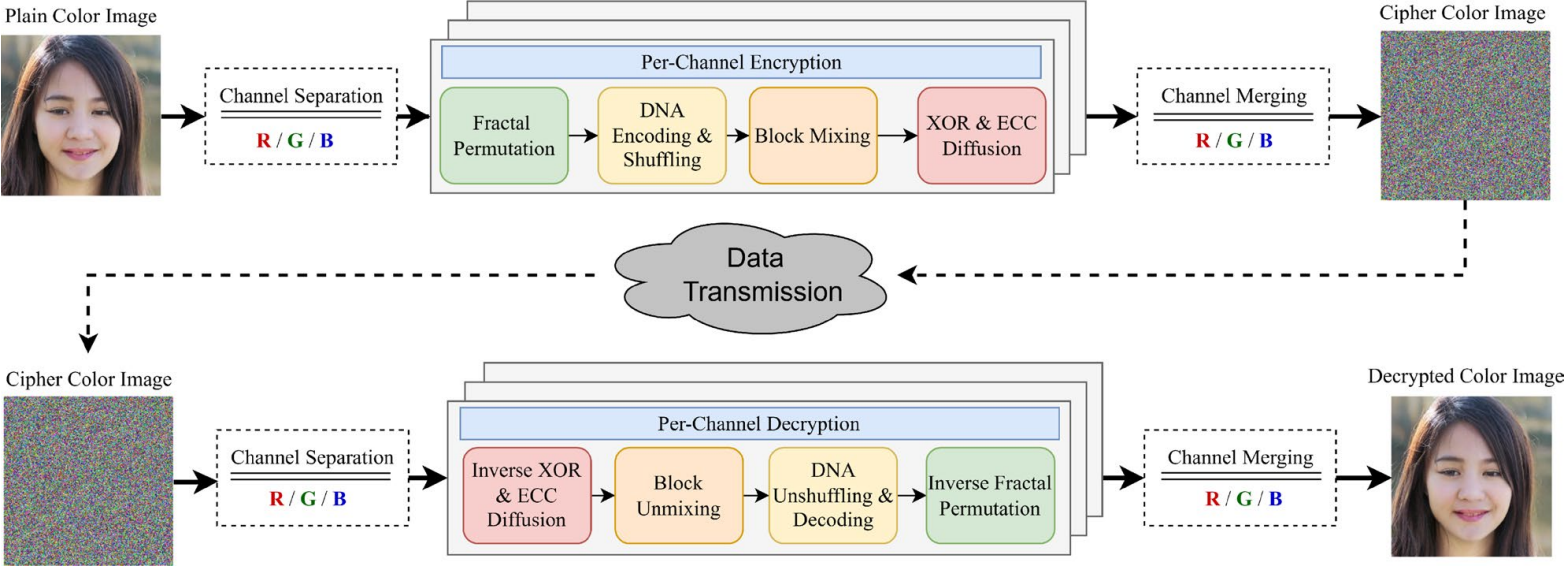
Functional overview for privacy-preserving image encryption pipeline.

A lossless multilayer color image encryption system is used to secure the images while they are uploaded to the cloud storage. It ensures security and its structure. Although every cloud platform has its own designs for security, using it adds another layer of security even if the approach of the relying party fails^49^. This study uses a combination of chaos theory, fractal permutation, DNA-based encoding, matrix-based block mixing, and elliptic-curve-based diffusion. The proposed scheme works independently on each channel. The high-level functional view of the approach is presented in Fig. 4. However, they share a secret key derived from a user-defined password.

Let *P* denote the user-defined secret password. A 256-bit cryptographic hash is generated using the Secure Hash Algorithm (SHA)-256 algorithm. The hash output is divided into multiple segments to derive the initial parameters of the chaotic system. These parameters are used to define a coupled chaotic map. The final chaotic sequence is presented in Eq. (2).

2 $$\begin{aligned} C_n = \frac{x_n + y_n}{2}, \quad n = 1,2,\dots ,N \end{aligned}$$

where *N* denotes the required sequence length based on image size, $x_n$, and $y_n$ represents the parameters for the coupled contributions.

Given an input color image $I \in \mathbb {Z}^{H \times W \times 3}$, it is decomposed into three independent channels:

3 $$\begin{aligned} I = \{I_R, I_G, I_B\} \end{aligned}$$

Each channel is encrypted separately using the same cryptographic parameters.

The initial layer uses a Mandelbrot-set-based fractal permutation to achieve spatial confusion. The escape time for each pixel coordinate is calculated using Eq. (4).

4 $$\begin{aligned} Z_{n+1} = Z_n^2 + C, \quad Z_0 = 0 \end{aligned}$$

The pixels are sorted according to the escape iteration counts. It produces a permutation vector *π*. Each pixel value is converted into a DNA sequence by linking its binary representation to four nucleotide bases. A predefined DNA mapping rule is as: $00 \rightarrow A,\quad 01 \rightarrow T,\quad 10 \rightarrow G,\quad 11 \rightarrow C$. A chaotic sequence is used to introduce additional confusion.

The shuffled image is divided into non-overlapping *2 × 2* blocks. For each block, a unimodular matrix *M* is constructed using a chaos-derived parameter. The block transformation is performed using Eq. (5).

5 $$\begin{aligned} B' = M \cdot B \cdot M^T \mod 256 \end{aligned}$$

This step provides strong diffusion for local pixel neighborhoods.

A key stream $K_{ECC}$ is generated using Elliptic-Curve–Cryptography (ECC)-inspired hashing and pseudo-randomization. ECC is also used here to share the key. It is used to increase security. The final encrypted pixel values are computed using Eq. (6).

6 $$\begin{aligned} E = B' \oplus K_{ECC} \end{aligned}$$

The same process in reverse will result in the decryption using the same secret key. XOR diffusion is first removed, followed by inverse matrix unmixing using $M^{-1}$, DNA reshuffling and decoding, and inverse fractal permutation. In the end, all decrypted RGB channels are merged to recover the original color image without loss.

## Experimental details and results analysis

This section highlights the details of the simulations and experiments conducted to evaluate the efficacy of the proposed approach. It analyzes the results achieved.

### Simulation setup

The proposed approach was evaluated on Google Colab Pro and NVIDIA A100 GPU. Various libraries were used to design and simulate the results, including TensorFlow 2.18.0 with Keras API, NumPy, etc. Fixed random seeds were selected for reproducibility. Each model undergoes independent training with the same data splits and preprocessing pipelines. The batch size is set to 64 samples per batch, and the training runs for a maximum of 20 epochs. The Adam optimizer with model-specific learning rates $\alpha = 1 \times 10^{-4}$ was used for training. In addition, various callbacks and regularization methods are used during training to improve the model:

- Early stopping: Training stops if there is no improvement in validation accuracy for 7 consecutive epochs.
- Learning rate scheduling: ReduceLROnPlateau with factor 0.2 and patience 4 was used.
- Dropout regularization: Applied progressively in FC layers.
- Model checkpointing: Best weights saved based on minimized validation losses.

### Performance metrics

As the problem statement involves binary classification, various classification performance measures are used. It includes accuracy, precision, recall, etc. In addition, various metrics are used to evaluate the computational cost and evaluate the efficacy of the model.

### Results analysis of the proposed SMF model

**Fig. 5:**
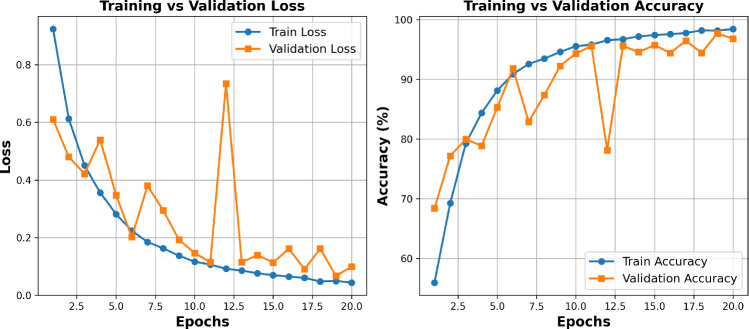
Visualization of trends in accuracy vs. loss during training.

Figure 5 visualizes trends in loss and accuracy for the proposed model. During the early stages of training, a sharp loss in loss was observed with an increase in accuracy. It was found to stabilize and move towards its ideal value. The accuracy curve was also observed to gradually converge. In addition, the close alignment between the training and validation curves suggests that the proposed SMF model achieves good generalization with minimal overfitting. A minor fluctuation in validation loss was observed due to batch variability and the inherent complexity of the features classified from the facial images.

**Fig. 6:**
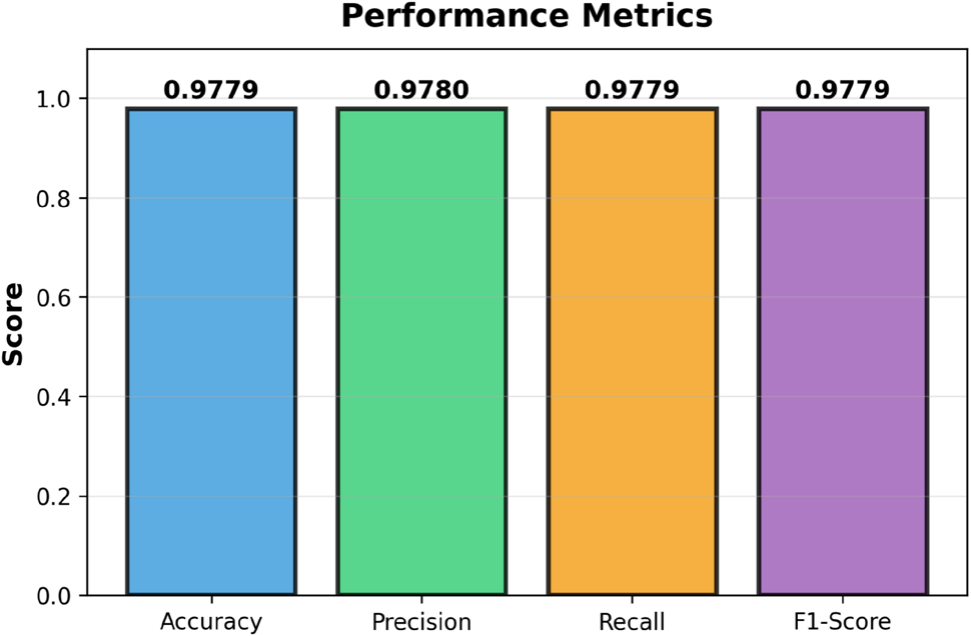
Visualization of different performance metrics for the proposed SMF model.

**Fig. 7:**
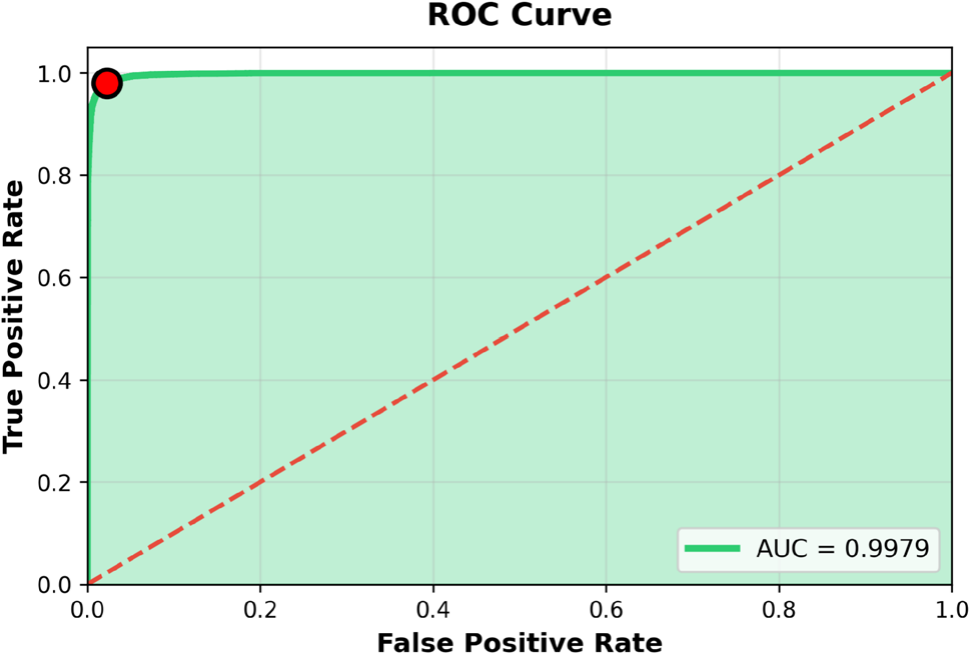
Visualization of the ROC curve for the proposed SMF model.

**Fig. 8:**
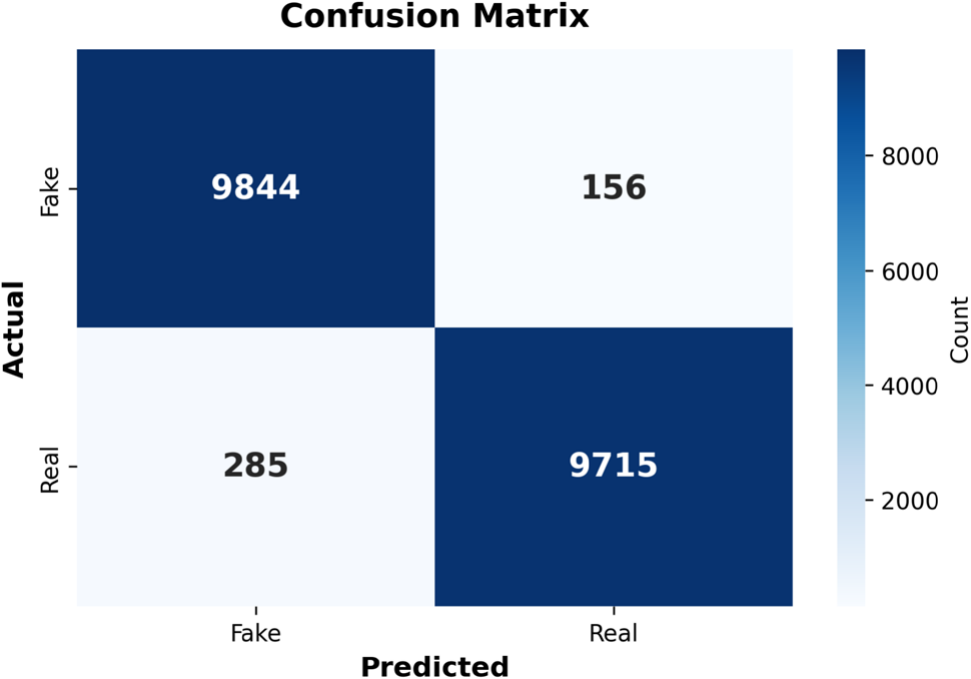
Confusion matrix of the proposed SMF model.

Figure 6 visualizes the overall results of the proposed SMF model with various performance metrics. It achieved a test accuracy of 97.79. In addition, the F1-score was observed to reach 97.8%. This balanced performance for all metrics indicates that the features learned by the model provide effective discrimination. In addition, the Receiver Operating Characteristic (ROC) curve of the proposed SMF model is presented in Fig. 7. The near-ideal value of 0.9979 demonstrates the ability to distinguish real and deepfake images for different decision thresholds. It is further supported by the confusion matrix in Fig. 8. The low error rate and high correct prediction indicate the effectiveness of the model to detect fine fake patterns.

**Fig. 9:**
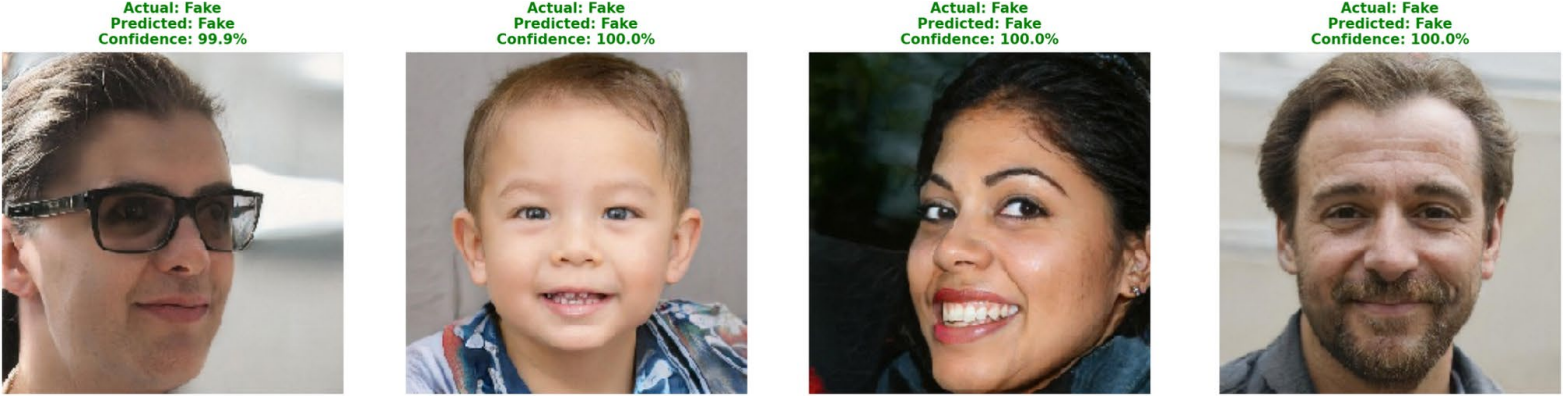
Sample test results of the proposed SMF model.

Figure 9 presents the results of the images randomly selected from the test set. In addition, a confidence score is evaluated to quantify the degree of correct prediction by the model. The score is based on the accuracy of the individual image during inference. It can be observed that the proposed SMF model correctly detects images with a high degree of confidence.

### Proposed SMF model compared to various PTMs

A comparative analysis against various PTMs has been conducted to evaluate its efficacy over them. They are trained under similar parameters for a fair comparison. The complexity of the model, the architectural design, and various other parameters are taken into account. It is conducted to benchmark the proposed SMF approach against various established models. It demonstrates the effectiveness of the proposed SMF model. The results are summarized in Table 4. It highlights total parameters (including optimizer parameters), trainable parameters, non-trainable parameters, etc.

**Table 4:** Parameter analysis of different PTMs with proposed SMF model.

Model name	Model type		Total parameters	Trainable parameters	Non-trainable parameters	Optimizer parameters	Model size (MB)
	Pre-trained	Scratch					
VGG16	*\checkmark*		15,704,520	329,602	14,715,712	659,206	59.91
VGG19	*\checkmark*		21,014,216	329,602	20,025,408	659,206	80.16
Inceptionv3	*\checkmark*		25,151,912	1,116,034	21,803,808	2,232,070	95.95
ResNet50	*\checkmark*		26,936,840	1,116,034	23,588,736	2,232,070	102.76
MobileNetv2	*\checkmark*		4,427,464	722,818	2,259,008	1,445,638	16.89
**Proposed SMF Model**		*\checkmark*	**82,903,248**	**27,629,082**	**16,000**	**55,258,166**	**316.25**

**Table 5:** Performance comparison of different PTMs with proposed SMF Model.

Model name	Best model epoch	Best training accuracy (%)	Best training loss	Best validation accuracy (%)	Best validation loss	Test accuracy (%)	Test loss
VGG16	20	78.27 (ep. 20)	0.4615 (ep. 20)	81.96 (ep. 20)	0.4042 (ep. 20)	81.75	0.4060
VGG19	18	76.94 (ep. 19)	0.4830 (ep. 19)	79.37 (ep. 18)	0.4424 (ep. 18)	79.34	0.4480
Inceptionv3	20	79.44 (ep. 20)	0.4419 (ep. 19)	82.06 (ep. 20)	0.4018 (ep. 20)	81.95	0.4049
ResNet50	13	66.35 (ep. 15)	0.6137 (ep. 15)	68.36 (ep. 13)	0.5892 (ep. 13)	68.84	0.5920
MobileNetv2	12	81.95 (ep. 14)	0.3992 (ep. 14)	83.29 (ep. 12)	0.3727 (ep. 12)	83.31	0.3750
**Proposed SMF Model**	**19**	**98.44 (ep. 20)**	**0.0436 (ep. 20)**	**97.71 (ep. 19)**	**0.0670 (ep. 19)**	**97.79**	**0.0680**

The PTMs rely on transfer learning, where most layers remain non-trainable. It increases the total number of parameters, but a large number of them are non-trainable. Although this approach is effective for the usual image recognition task, it reduces the adaptability of the model to detect deepfake artifacts. On the other hand, the proposed SMF model is trained from scratch. It increases the total number of trainable parameters as compared to non-trainable ones. It has the lowest non-trainable parameters and the highest trainable parameters among all. It increases the overall performance of the model, as illustrated in Table 5. The accuracies of the PTMs ranged from 68.84%–83.31%. On the other hand, the proposed SMF model achieved a test accuracy of 97.79%. It represents an improvement of 14.5% and 29% over the best and weakest PTMs. In addition, the lowest test loss of 0.0680 among all indicates effective training and realization of patterns.

**Fig. 10:**
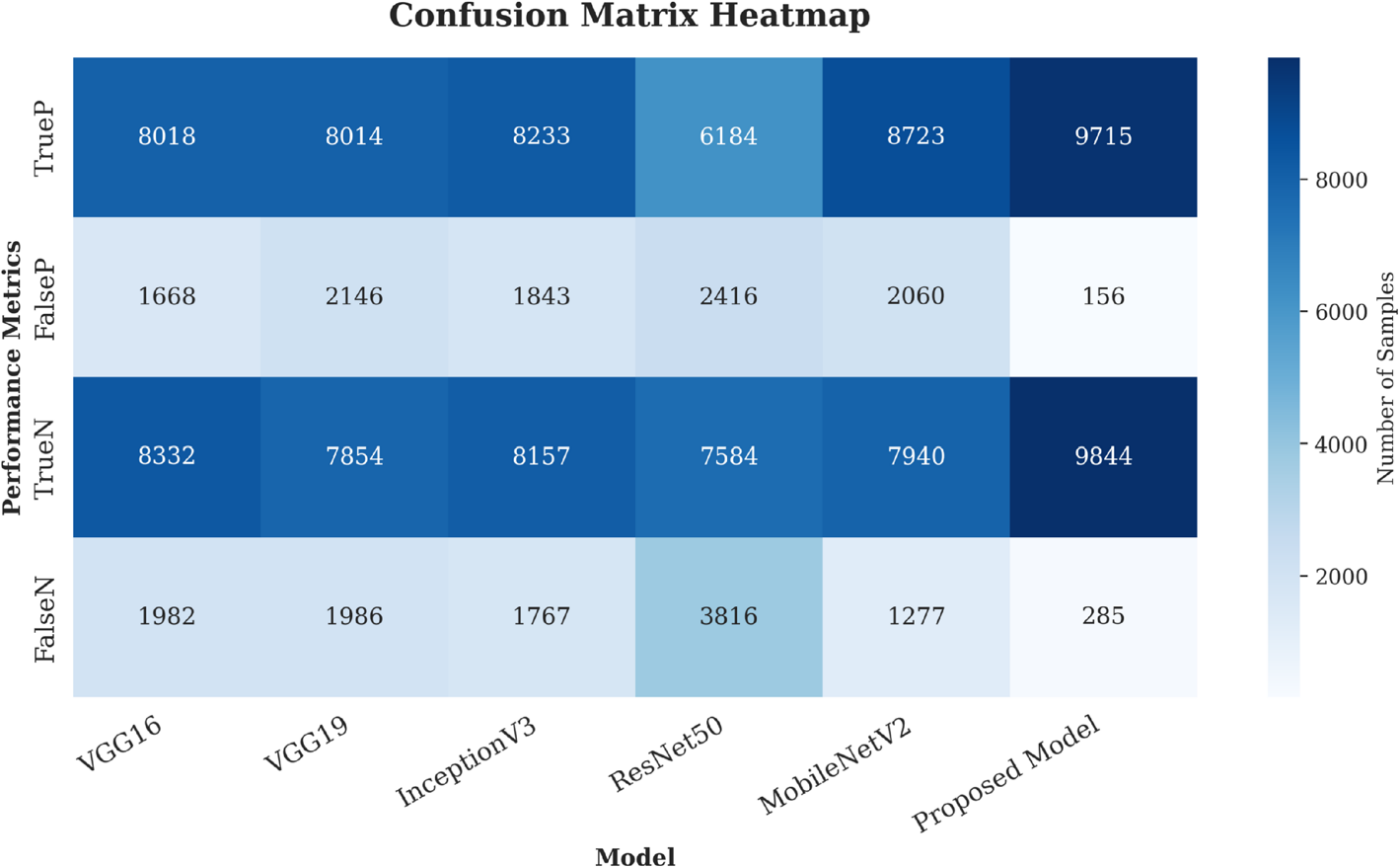
Summarization of the confusion matrix for different PTMs.

The detailed class-wise analysis of different metrics for PTMs is presented in Table 6. The F1-score of the PTMs ranged between 0.66–0.84. It represents inconsistent sensitivity for both classes. In addition, the ROC-AUC score of <0.92 indicates weaker classification in different decision thresholds. On the other hand, the improvement of 7–12% and 8% in the F1-scores and the ROC-AUC represents a high and consistent performance. It is further supported by the confusion matrix in Fig. 10. The proposed SMF model achieved a reduction of more than 85% in misclassification errors compared to various baseline models. This substantial reduction represents unbiased learning towards either class.

**Table 6:** Classification results of different PTMs with proposed SMF model.

Model name	Precision		Recall		F1-Score		Specificity	ROC-AUC
	Real	Fake	Real	Fake	Real	Fake		
VGG16	0.8278	0.8078	0.8018	0.8332	0.8146	0.8203	0.8332	0.8993
VGG19	0.7888	0.7982	0.8014	0.7854	0.7950	0.7917	0.7854	0.8779
InceptionV3	0.8171	0.8219	0.8233	0.8157	0.8202	0.8188	0.8157	0.8998
ResNet50	0.7191	0.6653	0.6184	0.7584	0.6649	0.7088	0.6184	0.7608
MobileNetV2	0.8090	0.8615	0.8723	0.7940	0.8394	0.8264	0.7940	0.9196
**Proposed SMF Model**	**0.9842**	**0.9719**	**0.9715**	**0.9844**	**0.9778**	**0.9781**	**0.9844**	**0.9979**

**Fig. 11:**
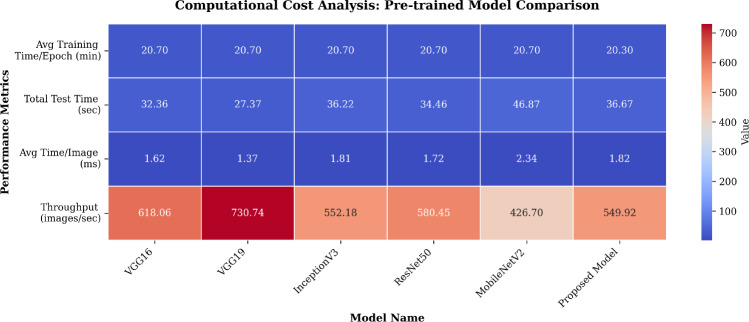
Comparison of computational time of different PTMs with the proposed SMF model.

The computational cost analysis of PTMs is shown in Fig. 11. It highlights different metrics to evaluate the real-life deployment of the models. The proposed SMF model achieved a comparable inference time with InceptionV3 and ResNet50, despite having more parameters. The high throughput of *∼*549.2 images/s with an average inference time of 1.82 ms indicates a decent practical deployment scenario. Although a few models achieved slightly high throughput with reduced inference time, their trade-off between performance and computational cost is too high. It is also supported by the metrics for computational complexity, presented in Table 7. Although MobileNetV2 requires fewer parameters with a low model size, it also faces a high trade-off in performance metrics. This makes such a model inefficient for performance-critical scenarios but suitable for scenarios that can cope with this trade-off in performance and have a limited computational environment. The proposed SMF model achieved a low Floating Point Operations (FLOPs) in gigabytes and Giga Multiply–Accumulate Operations (GMACs) as compared to other models. It indicates the computational feasibility of the approach over SOTA PTMs. In addition, a competitive low FLOPs per parameter and high parameters per GFLOP (in thousands) indicate the parameter efficiency of the model. The proposed SMF model achieved high performance compared to various standard PTMs with a slight trade-off in computational cost metrics.

**Table 7:** Computational Complexity Comparison of Proposed SMF model with SOTA models.

Model	Params	Size (MB)	GFLOPs	GMACs	FLOPs/Param	Params/GFLOP (K)
VGG16	15,045,314	57.39	30.7136	15.3568	2041.41	489.86
VGG19	20,355,010	77.65	39.0385	19.5193	1917.88	521.41
InceptionV3	22,919,842	87.43	5.6956	2.8478	248.50	4024.14
ResNet50	24,704,770	94.24	7.7534	3.8767	313.84	3186.30
MobileNetV2	2,981,826	11.37	0.6142	0.3071	205.97	4855.02
**Proposed Model**	**27,645,082**	**105.46**	**7.8595**	**3.9297**	**284.30**	**3517.41**

### Ablation study

An ablation study is performed to analyze the contribution of each component of the proposed SMF model. They are tested under the same environmental setup for a fair evaluation. They were systematically added, and their performance was evaluated. As a result, the individual strength of the components could be tested. The variants (Var) tested in the approach are further termed as follows:

#### Var-1: baseline CNN model

The traditional CNN structure acts as a foundational architecture to benchmark performance. This design follows Eq. (7) for computation:

7 $$\begin{aligned} \begin{aligned} f_{\text {baseline}}(x) =\;&\text {Dense}_{\text {out}}\Big (\text {Dropout}_{0.3}\big (\text {Dense}_{128}^{\text {ReLU}}\big (\text {Dropout}_{0.5}\big (\text {Dense}_{512}^{\text {ReLU}}\big ( \\&\text {Flatten}\big (\text {ConvBlock}_4\big (\text {ConvBlock}_3\big (\text {ConvBlock}_2\big (\text {ConvBlock}_1(x) \big )\big )\big )\big )\big )\big )\Big ) \end{aligned} \end{aligned}$$

where each $\text {ConvBlock}_i$ consists of:

$$\begin{aligned} \text {ConvBlock}_i(x) = \text {MaxPool} \text {Conv2D}_{f_i}^{\text {ReLU},\,3\times 3,\,\text {same}}(x))\\ f_i&\in \{32, 64, 128, 256\} \text { for } i \in \{1, 2, 3, 4\} \end{aligned}$$

The architecture consists of two FC layers and an output layer, as mentioned subsequently:

$$\begin{aligned} \text {FC}_1&: 512 \text { neurons with ReLU activation and 50\% dropout} \\ \text {FC}_2&: 128 \text { neurons with ReLU activation and 30\% dropout} \\ \text {Output}&: 2 \text { neurons with softmax activation} \end{aligned}$$

This baseline architecture uses standard convolutional feature extraction without advanced normalization or attention mechanisms. The progressive filter increase (*32 → 256*) enables hierarchical feature learning from low-level edges to high-level semantic representations.

#### Var-2: batch-normalized CNN model

The second architecture investigates the impact of BN for DFD. BN addresses internal covariate shifts and potentially improves the model’s ability to learn stable feature representations. It allows the model to learn more discriminative features to distinguish real from synthetic faces. The architectural design follows Eq. (8) for computation:

8 $$\begin{aligned} \begin{aligned} f_{\text {batchnorm}}(x) =\;&\text {Dense}_{\text {out}}\big (\text {Drop}_{0.3}\big (\text {Dense}_{128}\big ( \\&\text {Drop}_{0.5}\big (\text {BN}\big (\text {Dense}_{512}\big (\text {Flatten}\big (\text {BNConvBlock}_4\big (\text {BNConvBlock}_3\big ( \\&\text {BNConvBlock}_2\big (\text {BNConvBlock}_1(x) \big )\big )\big )\big )\big )\big )\big )\big ) \end{aligned} \end{aligned}$$

where each batch-normalized convolutional block is defined using Eqs. (9) and (10):

9 $$\begin{aligned} \text {BNConvBlock}_i(x)&= \text {MaxPool}(\text {BN}(\text {ReLU}(\text {Conv2D}_{f_i}(x)))) \end{aligned}$$

10 $$\begin{aligned} \text {BN}(x)&= \gamma \frac{x - \mu _B}{\sqrt{\sigma _B^2 + \epsilon }} + \beta \end{aligned}$$

Where $\mu _B$ and $\sigma _B^2$ represent the mean and variance of the batch. On the other hand, *γ* and *β* are learnable parameters. *ε* is a small constant for numerical stability.

#### Var-3: SE-attention enhanced CNN model

The third architecture uses SE attention mechanisms and residual connections to improve the model’s ability. Attention mechanisms enable the model to adaptively weight different spatial and channel features. The residual connections facilitate gradient flow and enable deeper networks. In addition, Global Average Pooling (GAP) provides a better generalization compared to flattening operations. It helps to focus on critical facial regions that may contain synthetic artifacts. The architectural configuration of the model is presented in Eq. (11).

11 $$\begin{aligned} f_{\text {attention}}(x)&= \text {Dense}_{\text {out}}(\text {Drop}_{0.3}(\text {Dense}_{128}(\text {Drop}_{0.5}(\text {BN}(\text {Dense}_{512} \nonumber \\&\quad (\text {GAP}(\text {MaxPool}(\text {ResBlock}(\text {SEConvBlock}_3(\text {SEConvBlock}_2 \nonumber \\&\quad (\text {SEConvBlock}_1(x))\big )\big )\big )\big )\big )\big )\big )\big )\big )\big ) \end{aligned}$$

The SE block is mathematically defined as:

$$\begin{aligned} \text {SE}(X)&= X \odot \sigma (\text {FC}_2(\delta (\text {FC}_1(\text {GAP}(X))))) \\ \text {GAP}(X)&= \frac{1}{H \times W} \sum _{i=1}^{H} \sum _{j=1}^{W} X_{i,j} \\ \text {FC}_1&: \mathbb {R}^C \rightarrow \mathbb {R}^{C/r} \\ \text {FC}_2&: \mathbb {R}^{C/r} \rightarrow \mathbb {R}^C \end{aligned}$$

where *⊙* denotes element-wise multiplication, *σ* represents sigmoid function, *δ* represents ReLU activation, and *r* signifies the reduction ratio (set to 16). In addition, *C*, *H*, and *W* represent channel, height, and width dimensions, respectively.

The enhanced residual block incorporates SE attention, as mentioned in Eq. (12).

12 $$\begin{aligned} \begin{aligned} \text {ResBlock}(x)&= \text {ReLU}(\text {SE}(\text {BN}(\text {Conv2D}(\text {BN}(\text {ReLU}(\text {Conv2D}(x)))))) + \text {BN}(\text {Conv2D}_{1\times 1}(x))) \end{aligned} \end{aligned}$$

#### Var-4: proposed SMF model

The proposed architecture represents the most sophisticated design among the ones mentioned. It integrates their advanced techniques, including multi-scale feature fusion and enhanced residual blocks. The dual-branch approach combines GAP for global context with localized feature extraction. The design follows Eq. (13) for computation:

13 $$\begin{aligned} f_{enhanced}(x) = \text {MLP}(\text {Concat}(\text {Branch}_1(F), \text {Branch}_2(F))) \end{aligned}$$

where *F* represents the high-level feature maps obtained using Eqs. (14) and (15):

14 $$\begin{aligned} \begin{aligned} F&= \text {EnhancedResBlock}_3(\text {MaxPool}_{2\times 2}(\text {EnhancedResBlock}_2(\text {EnhancedResBlock}_1( \\&\quad \text {SEConvBlock}_2(\text {SEConvBlock}_1(\text {InitialBlock}(x))))))) \end{aligned} \end{aligned}$$

15 $$\begin{aligned} \text {InitialBlock}(x)&= \text {MaxPool}_{3 \times 3}(\text {BN}(\text {ReLU}(\text {Conv2D}_{7 \times 7, s=2}(x))\big )\big ) \end{aligned}$$

The multi-scale feature fusion branches can be defined using Eqs. (16) and (17):

16 $$\begin{aligned} \text {Branch}_1(F)&= \text {Dense}_{256}(\text {GAP}(F)) \end{aligned}$$

17 $$\begin{aligned} \text {Branch}_2(F)&= \text {Dense}_{256}(\text {Flatten}(\text {MaxPool}_{2 \times 2}(F))\big ) \end{aligned}$$

The final classification head processes the concatenated features using Eq. (18).

18 $$\begin{aligned} \begin{aligned} \text {MLP}(x)&= \text {Softmax}(\text {Dense}_2(\text {Drop}_{0.3}(\text {Dense}_{128}(\text {Drop}_{0.4}(\text {BN}(\text {Dense}_{512}(\text {Drop}_{0.5}(\text {BN}(x)\big )\big )\big )\big )\big )\big )\big )\big ) \end{aligned} \end{aligned}$$

**Table 8:** Parameter analysis of the ablated versions.

Variant	Total parameters	Trainable parameters	Non-trainable parameters	Optimizer parameters	Model size (MB)
Var-1	78,434,888	26,144,962	0	52,289,926	299.21
Var-2	78,442,824	26,146,946	1,984	52,293,894	299.24
Var-3	15,593,788	5,195,966	5,888	10,391,934	59.49
Var-4	82,903,248	27,629,082	16,000	55,258,166	316.25

**Table 9:** Comparison of computational time of the ablated versions.

Variant	Best model epoch	Best training accuracy (%)	Best training loss	Best validation accuracy (%)	Best validation loss	Test accuracy (%)	Test loss
Var-1	20	96.34 (ep. 20)	0.0961 (ep. 20)	96.44 (ep. 20)	0.0959 (ep. 20)	96.36	0.0960
Var-2	16	97.47 (ep. 20)	0.0681 (ep. 20)	96.49 (ep. 16)	0.0875 (ep. 16)	96.75	0.0880
Var-3	12	99.27 (ep. 19)	0.0197 (ep. 19)	96.27 (ep. 12)	0.1097 (ep. 12)	96.33	0.1100
Var-4	19	98.44 (ep. 20)	0.0436 (ep. 20)	97.71 (ep. 19)	0.0670 (ep. 19)	97.79	0.0680

The results for the ablated variants are presented in Tables 8 and 9. Var-1 and Var-2 were observed to generate a near-similar number of trainable parameters and model size despite adding batch normalization in the convolution layers. An additional non-trainable parameter of 1,984 was accounted for. The test accuracies for both the variants were found to be 96.36% and 96.75%, respectively. The improvement in Var-2 shows that normalization improves the training stability and feature representation quality with minimal overhead in parameters. On the other hand, changing the convolutional blocks with SE-convolution blocks resulted in a reduction of model size from 299 to 59 MB (*≈* 80% reduction). It is due to a reduction in trainable parameters due to its efficient design. It was observed to converge to the best accuracy by 12^th^ epochs as compared to other variants. However, there is a slightly large difference in training and validation accuracies (99.27% to 96.27%). In addition, the highest validation and test loss were observed for the model among the four variants. This degradation of performance in the attention model suggests that attention mechanisms alone require careful integration with complementary architectural components to achieve optimal performance. The proposed approach (Var-4) was observed to achieve the highest accuracies with minimal loss. It depicts stability as compared to other variants. This improvement shows that the combination of multiple architectural innovations produces synergistic effects that exceed the contributions of the individual components.

**Table 10:** Classification results of the ablated configurations.

Model name	Precision		Recall		F1-Score		Specificity	ROC-AUC
	Real	Fake	Real	Fake	Real	Fake		
Var-1	0.9566	0.9709	0.9713	0.9559	0.9639	0.9633	0.9559	0.9942
Var-2	0.9668	0.9681	0.9668	0.9681	0.9674	0.9675	0.9681	0.9951
Var-3	0.9951	0.9353	0.9311	0.9954	0.962	0.9644	0.9954	0.9984
Var-4	0.9842	0.9719	0.9715	0.9844	0.9778	0.9781	0.9844	0.9979

**Fig. 12:**
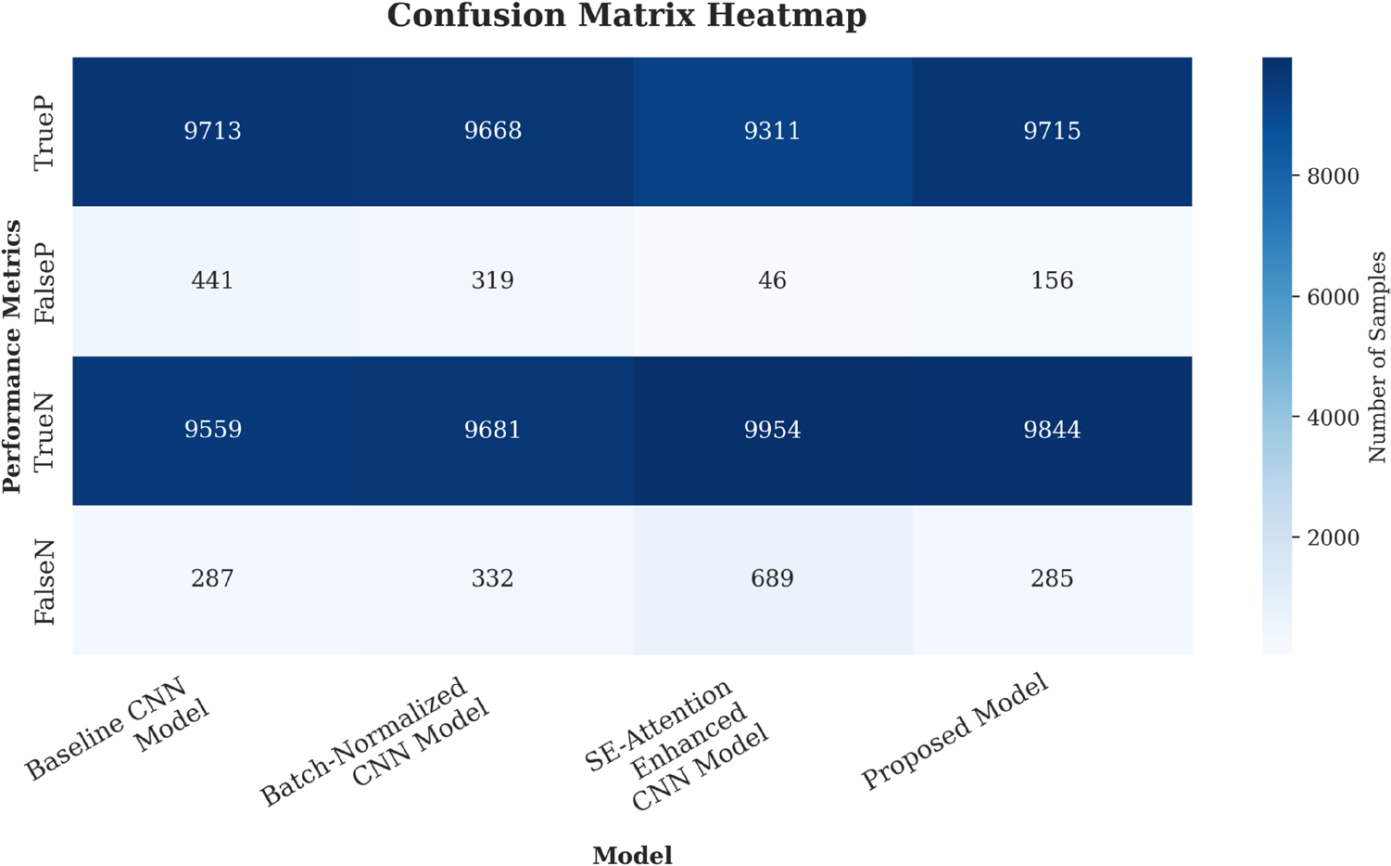
Summarization of the confusion matrix for different configurations.

**Fig. 13:**
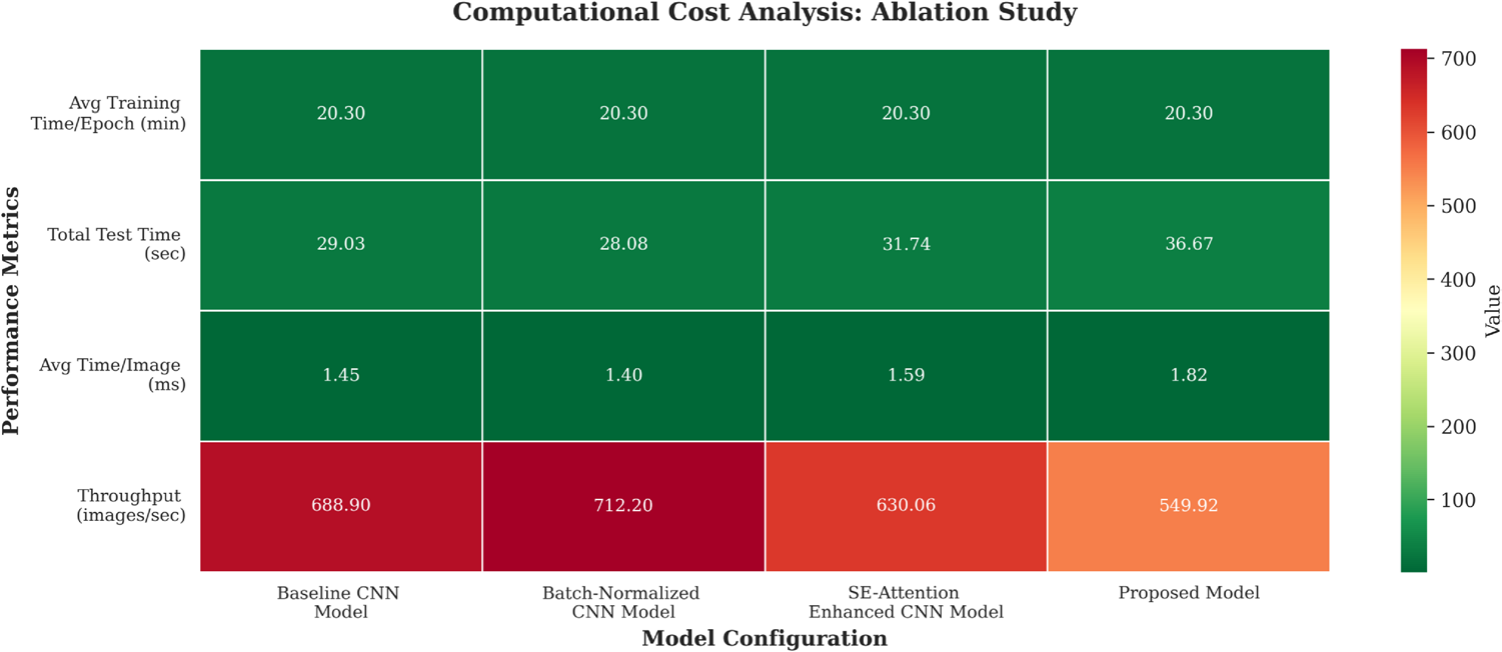
Comparison of computational time of ablated configuration of the SMF model.

The class-wise performance of the model and the observations are presented in Table 10 and Fig. 12. The proposed SMF model achieved various near-ideal metrics with an ROC-AUC score of 0.9979. This indicates strong class separability. However, ablated variants or configurations were observed to reduce various parameters, including F1-score by 1–2%. Although Var-3 achieves higher specificity and the ROC-AUC score, the reduction in recall indicates a trade-off between sensitivity and specificity. It was due to increased false negatives and reduced correct predictions. The results are further supported by the runtime performance ablation study in Fig. 13. Var-2 was found to achieve high throughput with reduced inference time. However, overall performance measures were reduced.

Based on the results of the ablation study, different variants or configurations of the proposed SFM model are suitable for different application scenarios depending on the accuracy requirements and computational constraints.

- Var-1: It is useful for low-latency or streaming inference scenarios where stable performance with small batch sizes is required.
- Var-2: It provides a balanced trade-off between performance and efficiency. This makes it appropriate for mid-scale deployments where both accuracy and computational time matter.
- Var-3: It is suitable for resource-constrained or real-time applications due to its smaller model size, faster inference, and higher throughput. For example, edge devices or mobile platforms.
- Var-4: It is ideal for high-computational environments where accuracy and robustness are prioritized over speed and model size. For example, cloud servers or offline forensic analysis

### Robustness analysis of the proposed SMF model

Robustness analysis is conducted to evaluate the stability of the proposed SMF model. It analyzes the performance of the proposed SMF model when subjected to images with various real-world manipulations. It ensures that the results are valid even if the image undergoes different geometric and photometric transformations. These attack vectors were chosen because they are commonly used in real-world image editing and social networks. The parameters for these attacks follow commonly adopted settings in the literature and ensure relevance to the real world^50,51^. Table 11 presents the performance of the proposed SMF model against seven different types of attacks. The *\checkmark* and *×* indicate correct and incorrect classification, respectively.

**Table 11:** Robustness analysis of the SMF model against various attacks.

Test sample name	Actual label	Attack type							
		Original (no attack)	Gaussian noise (*σ*=25)	JPEG compression (quality=30)	Image Resizing (50%)	Gaussian blur (7*×*7)	Brightness adjust (1.5*×*)	Contrast adjust (1.5*×*)	Zoom (1.2*×*)
ZYRKB9514S.jpg	Fake	*\checkmark*	*\checkmark*	*\checkmark*	*\checkmark*	*\checkmark*	*\checkmark*	*\checkmark*	*\checkmark*
2EQ7K91NID.jpg	Fake	*\checkmark*	*×*	*\checkmark*	*\checkmark*	*×*	*\checkmark*	*×*	*\checkmark*
4Q2SRFRTJG.jpg	Fake	*\checkmark*	*\checkmark*	*\checkmark*	*\checkmark*	*\checkmark*	*\checkmark*	*\checkmark*	*\checkmark*
XARHA6XT9Y.jpg	Fake	*\checkmark*	*\checkmark*	*\checkmark*	*\checkmark*	*×*	*\checkmark*	*×*	*\checkmark*
BFZ6Z7KEQ1.jpg	Fake	*\checkmark*	*\checkmark*	*\checkmark*	*\checkmark*	*\checkmark*	*\checkmark*	*\checkmark*	*\checkmark*
U88UQVYT9V.jpg	Fake	*\checkmark*	*\checkmark*	*\checkmark*	*\checkmark*	*\checkmark*	*×*	*\checkmark*	*\checkmark*
HBKY3K9D6B.jpg	Fake	*\checkmark*	*×*	*\checkmark*	*\checkmark*	*\checkmark*	*\checkmark*	*\checkmark*	*\checkmark*
09899.jpg	Real	*\checkmark*	*\checkmark*	*\checkmark*	*\checkmark*	*\checkmark*	*\checkmark*	*\checkmark*	*\checkmark*
3MOBZBAN67.jpg	Fake	*\checkmark*	*\checkmark*	*\checkmark*	*\checkmark*	*\checkmark*	*\checkmark*	*\checkmark*	*\checkmark*
35110.jpg	Real	*\checkmark*	*\checkmark*	*\checkmark*	*\checkmark*	*\checkmark*	*\checkmark*	*\checkmark*	*\checkmark*
10152.jpg	Real	*\checkmark*	*\checkmark*	*\checkmark*	*\checkmark*	*\checkmark*	*\checkmark*	*\checkmark*	*\checkmark*
6FY7HABJ2I.jpg	Fake	*\checkmark*	*\checkmark*	*\checkmark*	*\checkmark*	*\checkmark*	*\checkmark*	*\checkmark*	*\checkmark*
1336ARYN25.jpg	Fake	*\checkmark*	*\checkmark*	*\checkmark*	*\checkmark*	*\checkmark*	*\checkmark*	*\checkmark*	*\checkmark*
8TZM86XMW8.jpg	Fake	*×*	*\checkmark*	*×*	*×*	*×*	*×*	*×*	*×*
3SBDPBBSQE.jpg	Fake	*\checkmark*	*\checkmark*	*\checkmark*	*\checkmark*	*\checkmark*	*\checkmark*	*\checkmark*	*\checkmark*

**Fig. 14:**
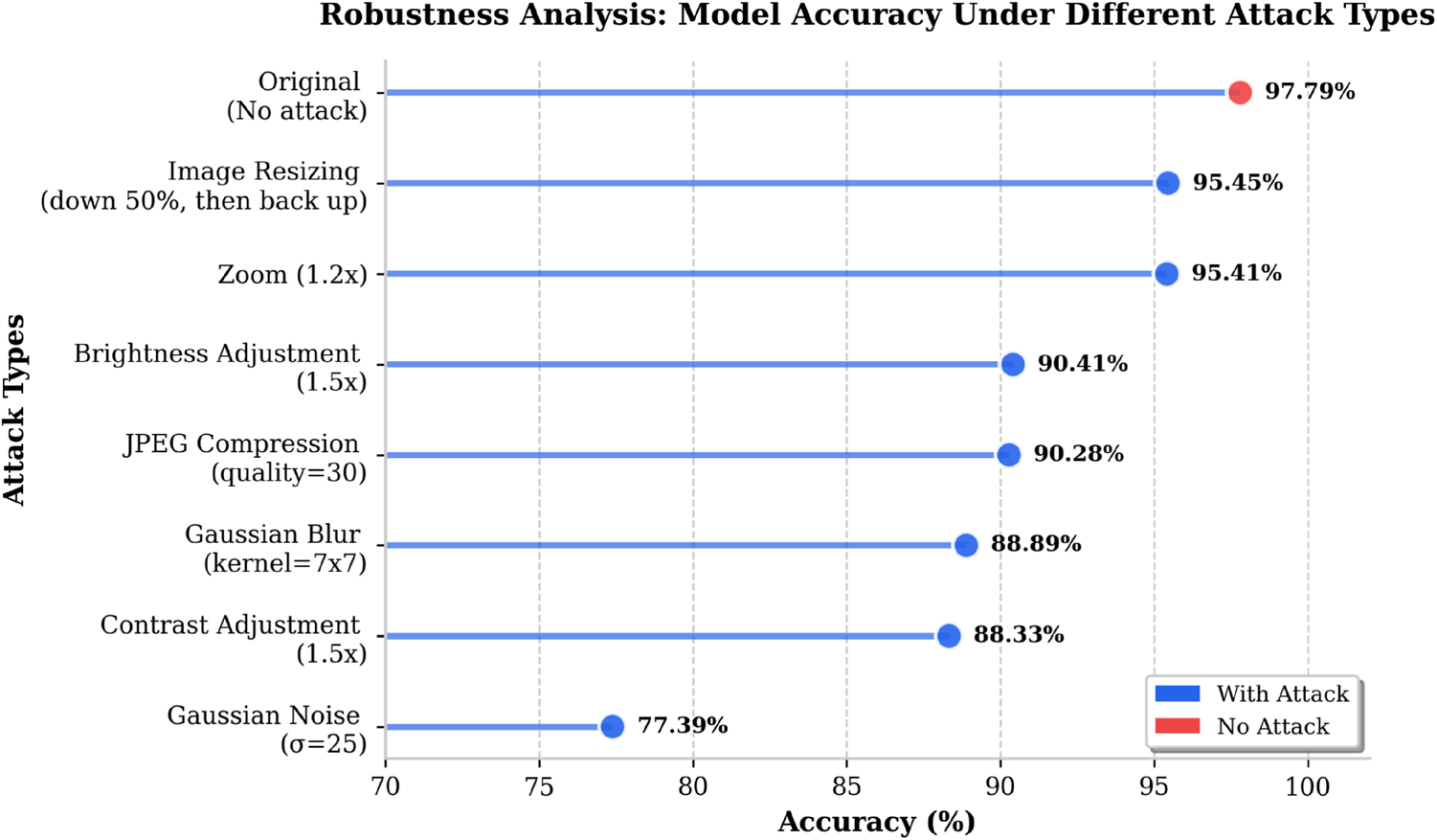
Visualization of trends in test accuracy of the proposed SMF model against different attack types.

**Fig. 15:**
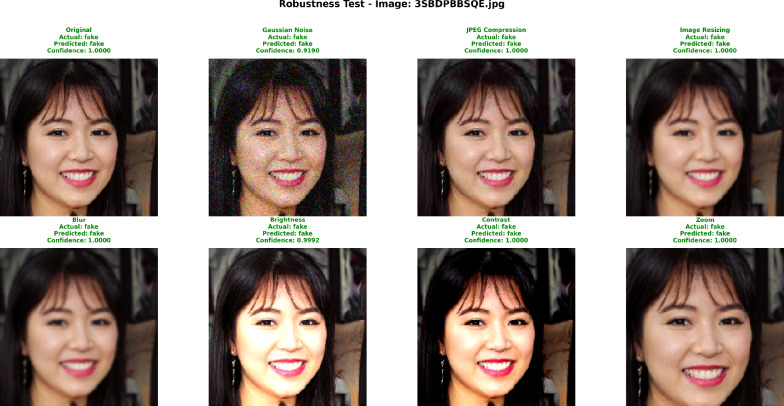
Robustness analysis of the proposed SMF model on a fake sample of the 140k RFF.

The proposed SMF model was observed to maintain the correct predictions against various transforms. It indicates that it relies on structural- and texture-based forgery artifacts. However, a few modifications, such as Gaussian noise, cause visual degradation in certain samples. They disrupt various high-frequency components and visual textures that are important for detecting fine fake artifacts. As a result, few fake samples were misclassified, as these disruptions suppress the features learned by the model. JPEG compression shows comparatively better robustness, as these artifacts still retain details of block-level inconsistencies used for detection. The trends for robustness based on test accuracy are summarized in Fig. 14. It shows the impact of different attacks on the classification accuracy. The proposed SMF model achieves high accuracy, followed by resizing, zooming, etc. However, a dire reduction can be observed for Gaussian noise. Figure 15 illustrate the test results of the real and fake image samples, respectively. The high and consistent scores for all the attacks indicate the robustness of the model against various image transformations. It was observed to be affected by a heavy injection of noise that interferes with texture-level forgery artifacts.

### Cross-dataset generalization of the proposed SMF model

A cross-dataset study is conducted to evaluate the generalization capability of the proposed SMF model. The capability of detecting unseen deepfakes correctly is an essential part of a model. Thus, the proposed SMF model is trained on a dataset and tested against five other unseen datasets. These datasets consist of various types of forgeries that are generated using different GANs and manipulation techniques. In addition, they are extensively used in existing studies. This provides a common benchmark for fair comparison. The datasets used are termed as follows:

- DS1: 140K RFF^10^
- DS2: deepfake and real images^52^
- DS3: CASIA v2^53^
- DS4: Deepfake-and-real-images^54^
- DS5: Real-and-fake-face-detection^55^
- DS6: Photoshopped-faces^56^

**Table 12:** Cross-dataset performance of the proposed SMF model.

Proposed SMF model	Cross dataset					
	DS1	DS2	DS3	DS4	DS5	DS6
Accuracy	0.9779	0.4934	0.4151	0.5066	0.4851	0.5045
Precision	0.9842	0.4948	0.4084	0.5052	0.4771	0.5023
Recall	0.9715	0.9841	0.9815	0.9902	0.9885	0.982
F1-Score	0.9778	0.6585	0.5768	0.669	0.6436	0.6646

The results are presented in Table 12. The proposed SMF model achieves consistent and effective performance on DS1. This shows that the model has learned strong and reliable features to detect synthetic faces. On the other hand, the model shows generalization issues when evaluated on the unseen datasets DS2–DS6. Metrics like accuracy and precision were observed to drop to *≈*0.41–0.51. It is observed as a generalizable weakness rather than a reasonable outcome. The recall was observed to remain high for all datasets. The combination of the resultant accuracy with high recall suggests the model tends to predict the fake class indiscriminately, rather than detecting forgeries through learned discriminative features. Since the model is trained solely on DS1, this reduced performance is due to the change in GAN architecture, data curation strategy, etc. It is mainly due to a domain shift. The fake images in other datasets are created using different techniques, compression levels, resolutions, post-processing operations, etc., that were not seen during training.

### Statistical significance of the proposed SMF

**Fig. 16:**
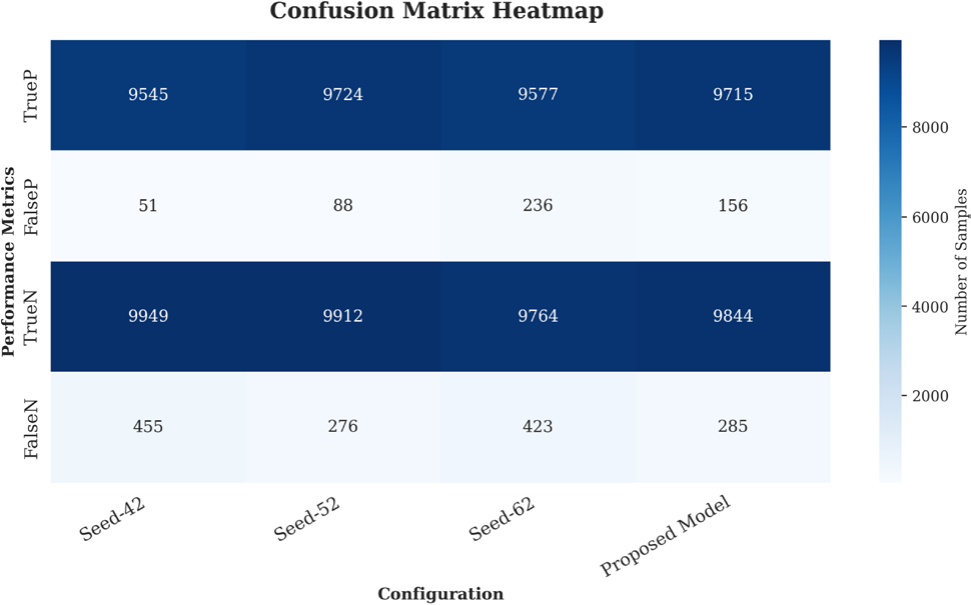
Confusion matrices of the proposed SMF model for different seeds.

In order to ensure that the proposed SMF model is not influenced by random initializations or data shuffle, a statistical stability analysis has been conducted. It involves the analysis of results for different random seeds. Two approaches are used throughout the training process. One consists of the option of shuffle=true, and the other uses random seeds for various purposes. It includes shuffling of training images, selection of neurons for dropout, and different random processes by the libraries used. Various metrics, including classification metrics, computational efficiency, etc., are presented in Tables 13 and 14. Figure 16 presents the confusion matrix of different seeds of the proposed SMF approach. It achieves a stable performance for all seed configurations. The test accuracy varies within a narrow range of 96.70%–98.18%. It indicates the robustness of the overall learning process against stochastic variations introduced by random weight initialization and data shuffling. The use of 52 as seed presented a test accuracy greater by 0.39% than the random shuffle of the proposed SMF model. It achieves the most balanced distribution with the least error. On the other hand, the one with 62 as a seed reported a loss in accuracy of 1.09%. This suggests convergence to a sub-optimal local minima.

**Table 13:** Performance comparison of the proposed SMF model with different seeds.

Configuration	Best Model epoch	Best training accuracy (%)	Best training loss	Best validation accuracy (%)	Best validation loss	Test accuracy (%)	Test loss
Seed=42	19	99.24 (ep. 20)	0.0217 (ep. 20)	97.46 (ep. 19)	0.0741 (ep. 19)	97.47	0.0750
Seed=52	17	99.05 (ep. 20)	0.0273 (ep. 20)	98.05 (ep. 17)	0.0535 (ep. 17)	98.18	0.0540
Seed=62	15	97.27 (ep. 15)	0.0726 (ep. 15)	96.68 (ep. 15)	0.0896 (ep. 15)	96.70	0.0900
Proposed SMF Model	19	98.44 (ep. 20)	0.0436 (ep. 20)	97.71 (ep. 19)	0.0670 (ep. 19)	97.79	0.0680
**Mean **±** Std**	17.50 ± 1.91	98.50 ± 0.89	0.0413 ± 0.0228	97.48 ± 0.58	0.0711 ± 0.0150	97.54 ± 0.63	0.0718 ± 0.0150

**Table 14:** Classification metrics of the proposed SMF model for different seeds.

Configuration	Precision		Recall		F1-Score		Specificity	ROC-AUC
	Real	Fake	Real	Fake	Real	Fake		
Seed=42	0.9947	0.9563	0.9545	0.9949	0.9752	0.9742	0.9949	0.999
Seed=52	0.991	0.9729	0.9724	0.9912	0.9816	0.982	0.9912	0.9988
Seed=62	0.976	0.9585	0.9577	0.9764	0.9667	0.9674	0.9764	0.9952
Proposed SMF Model	0.9842	0.9719	0.9715	0.9844	0.9778	0.9781	0.9844	0.9979
**Mean **±** Std**	0.9865 ± 0.0082	0.9649 ± 0.0087	0.9640 ± 0.0093	0.9867 ± 0.0081	0.9753 ± 0.0063	0.9754 ± 0.0062	0.9867 ± 0.0081	0.9977 ± 0.0018

**Fig. 17:**
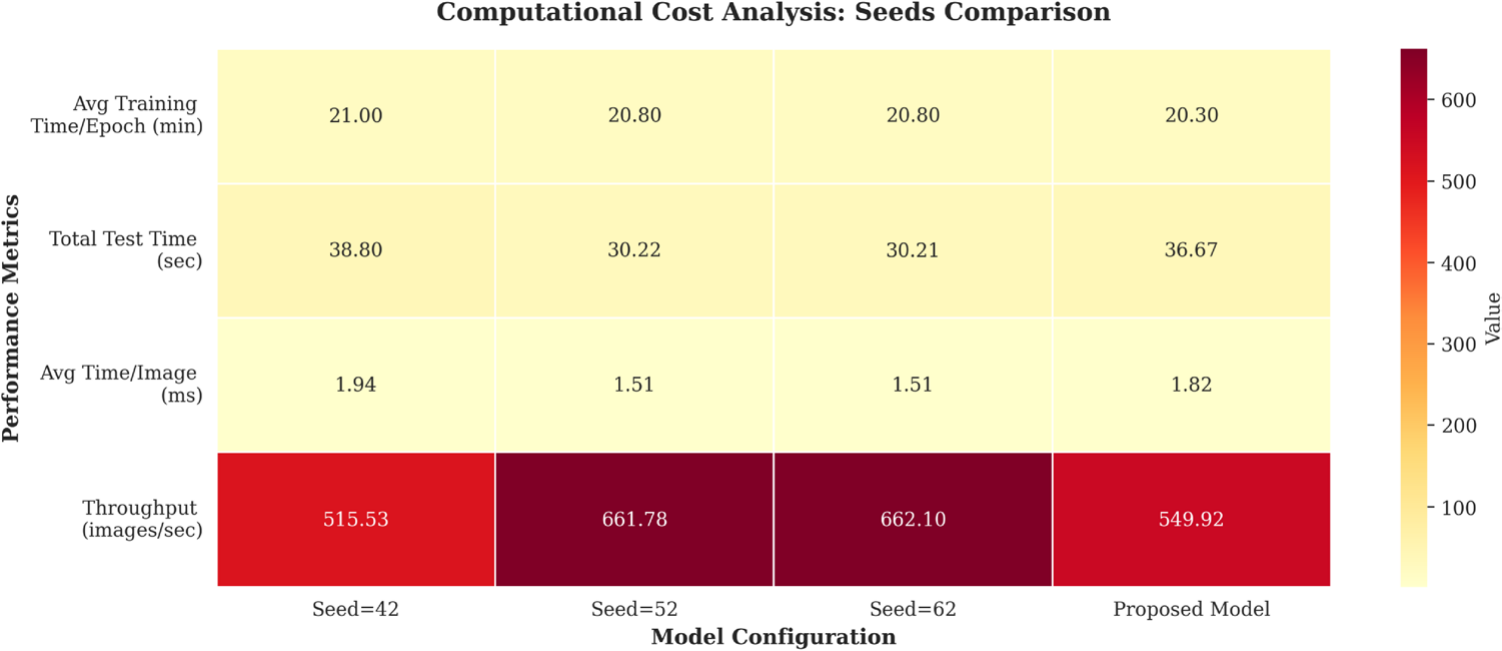
Computational cost analysis of the proposed SMF model for different seeds.

The computational stability of the proposed SMF model in the scenario of different seeds is presented in Fig. 17. The overall process was observed to be stable for all scenarios tested. The average training time per epoch remains constant at 20–21 minutes. This indicates deterministic computational complexity. Minor variations in the inference time are observed for different seeds. In addition, they were also observed to increase throughput to 661–662 images per second. On the other hand, 42 as a seed achieved slightly lower inference speed due to increased decision complexity, which is also presented in higher precision-based predictions. The low variance in accuracy and stable metrics, including ROC-AUC scores, indicates an efficient learning strategy.

### Privacy-preserving analysis

**Fig. 18:**
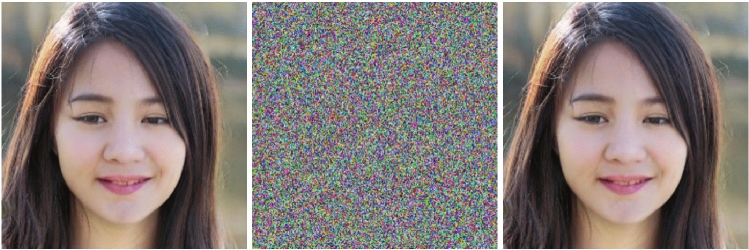
Visualization of real, encrypted, and decrypted images.

**Fig. 19:**
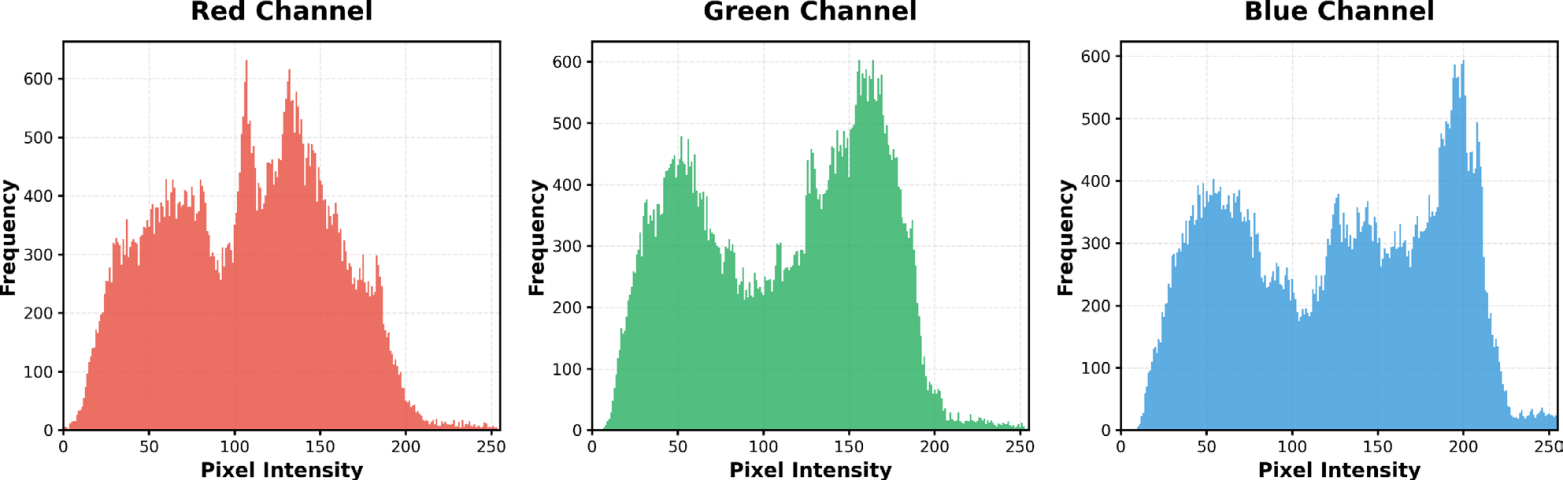
Visualization of RGB channel histograms for the original image.

**Fig. 20:**
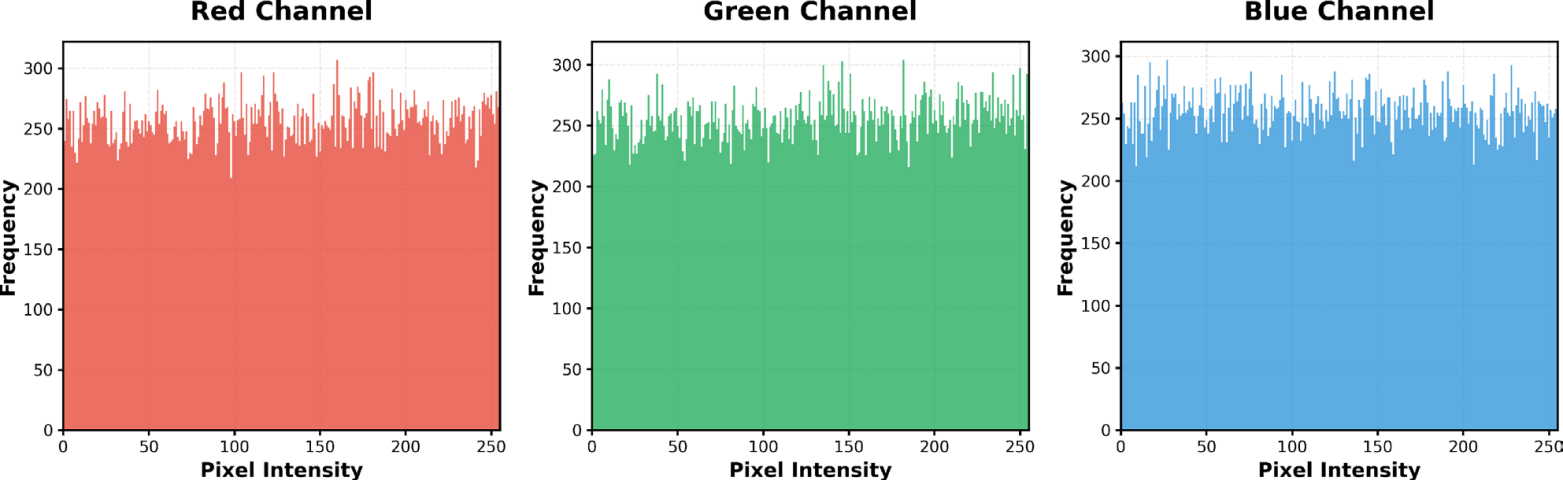
Visualization of RGB channel histogram for the encrypted image.

**Fig. 21:**
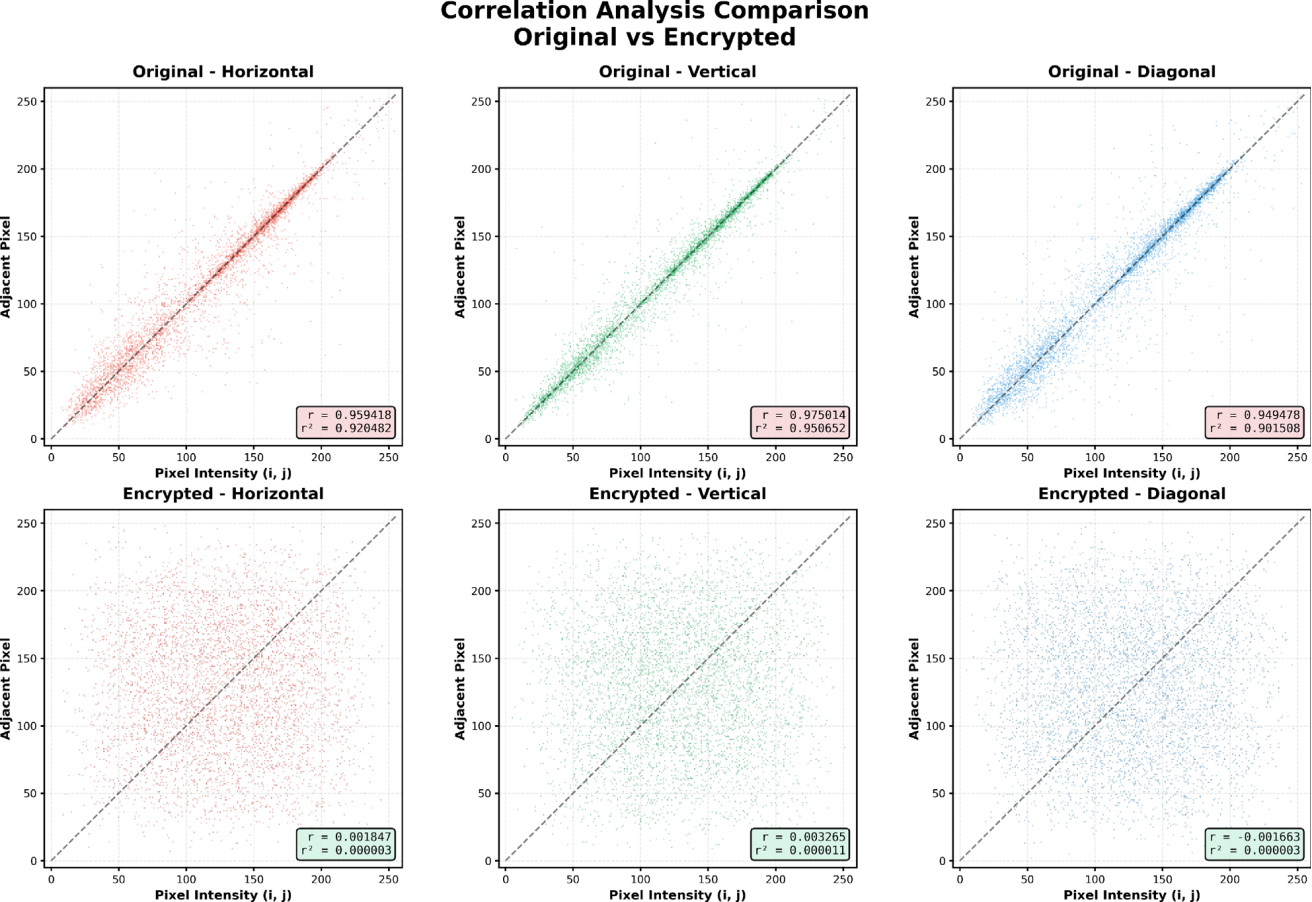
Pixel correlation analysis of original vs. encrypted images.

Figures 18, 19, 20 and 21 present a qualitative and statistical analysis of the results of image encryption. Their collective demonstration presents the effectiveness of the proposed encryption scheme in securing images and reducing statistical dependencies within the image.

Figure 18 presents the various stages of the cryptosystem. It presents a visual comparison between the original, encrypted, and decrypted image. The encrypted image was found to be visually unreadable to the naked human eye. The visible patterns and information are not readable in nature. In addition, there were no visual differences in the original and decrypted image. This suggests that there was no loss (Mean Squared Error (MSE) between the original and decrypted image) during the entire life-cycle.

Figures 19 and 20 present the histogram of the RGB channel for the original and encrypted images, respectively. The original image leaks out the values of each channel. This can help to understand the pixel intensities that may help to exploit them for statistical attacks. On the other hand, the histogram of the encrypted image appears as random noise. The histograms of all three channels were observed to be nearly uniform for the intensity range. This even distribution increases the difficulty for an attacker to extract statistical information. This behavior is highly recommended for a secure image encryption scheme.

The correlation analysis between adjacent pixels in three directions for both the original and encrypted images is depicted in Fig. 21. The strong correlations in the original images were observed. The scatter plots were observed to stay clustered along the diagonal. It shows high redundancy present in natural images. On the other hand, the scatter plots are dispersed. It shows that there is no visible correlation between the adjacent pixels. In addition, the correlation coefficients are also near-ideal to zero. It presents that the encryption process removes the dependencies in the pixels.

### Comparison of the proposed SMF model with existing related works

Table 15 presents the comparison among various existing architectures. The proposed SMF model achieves a high accuracy of 97.79% in the 140K RFF dataset. It is observed to outperform models built from scratch. On the other hand, the proposed SMF model achieves performance nearly comparable to the existing works. It is important to note that existing models often come with certain limitations. It includes high computational requirements, dependency on large external datasets, reduced flexibility for domain-specific adaptation, etc. However, the proposed SMF model, which is developed from scratch, offers a more efficient and adaptable approach for DFD. In addition, it can be indicated that semantic token approaches may focus on high-level semantic inconsistencies but struggle for DFD that maintain semantic coherence. The 8.83% gap in accuracy demonstrates the efficiency of DL-based approaches with architectural innovations.

**Table 15:** Performance metrics comparison of the proposed model against existing deepfake detection model.

Author(s)	Year	Model Name	Pre-trained	Dataset (Training)	Dataset (Testing)	Accuracy
Raj et al.^16^	2024	ELA + CNN	*×*	CASIA v2.0	CASIA v2.0	93.15%
Xu et al.^20^	2024	FAMSeC (Few-shot with LoRA + Contrastive)	*\checkmark*	ProGAN subset from ForenSynths, 0.56% of baseline	ForenSynths (7 GANs), UniversalFakeDetect (4 models), GenImage (6 diffusion models)	Average: 95.22%, ForenSynths: 96.52%, UniversalFake: 97.85%, GenImage: 90.90%
Biswas et al.^46^	2024	DenseNet169 + DenseNet201 Ensemble	*\checkmark*	CASIA 2.0	CASIA 2.0	93.79%
Alsolai et al.^22^	2025	Guardian-AI	*\checkmark*	CDDB	CDDB, ForenSynths, UniversalFakeDetect, GenImage	CDDB: 95.8%, ForenSynths: 96.52%, UniversalFake: 97.85%, GenImage: 90.90%
Peng et al.^26^	2025	Semantic Token Transformer (STT)	*\checkmark*	WildDeepfake, Frame sampling: every 24 frames	WildDeepfake, CelebDF-v2, FF++ (c23), DFDC	WildDeepfake: 88.96% , CelebDF-v2: 99.81%
Proposed SMF Model	2026	Stacked Multi-Fusion	*×*	140k RFF	140k RFF, Deepfake and real images, CASIA v2, Deepfake-and-real-images, Real-and-fake-face-detection, hotoshopped-faces	140k RFF: 97.79%, Deepfake and real images: 49.34%, CASIA v2: 41.51%, Deepfake-and-real-images: 50.66%, Real-and-fake-face-detection: 48.51%, hotoshopped-faces: 50.45%

### Comparison of the privacy-preserving model with existing models

Table 16 summarizes the performance of the proposed SMF model compared to various existing image cryptosystems. The SMF model was observed to achieve low correlation coefficients in all directions. Horizontal and diagonal correlations were observed to reduce by 45% and 81%, respectively, against Mehta et al.^57^. Although the researchers in Barik et al.^58^ report higher diagonal correlations, the SMF model maintains consistently low and ideal values in all directions. In addition, the proposed SMF model achieves a Number of Pixels Change Rate (NPCR) of 99.639%. It is 23% higher than Mehta et al.^57^ and comparable against various other state-of-the-art approaches. Although researchers in Barik et al.^58^ achieve a higher NPCR, the proposed SMF model achieves a lower Unified Averaged Changed Intensity (UACI) of 30.03%. It is nearly 10% lower than various existing methods. It indicates a stable variation of pixel intensities. The Information Entropy (IE) of 7.9999 is very close to the ideal value and presents a near-uniform distribution of the cipher image.

**Table 16:** Comparison of the proposed privacy-preserving model with existing methods.

Model	Correlation coefficient			NPCR	UACI	IE
	Horizontal	Vertical	Diagonal			
Mehta et al. ^57^	0.0033	0.0051	*-0.0091*	99.4123	49.7735	7.7475
Wang et al. ^59^	0.0009	0.0001	0.0019	99.6221	33.4509	7.9993
Barik et al. ^58^	*-0.0322*	0.0207	*-0.0503*	99.7900	33.4600	7.9998
Liu et al. ^60^	0.0007	0.0064	0.8098	99.6094	33.5062	7.9993
**Proposed Model**	0.0018	0.0033	*-0.0017*	99.6398	30.0260	7.9990

## Conclusion and future work

This paper presents a privacy-preserving deepfake image detection model for a complete life-cycle of an image. It proposes a CNN-based SMF model and a hybrid multilayer image cryptosystem with informed architectural choices for DFD. The proposed framework offers notable aspects and deeper insight into the building of sound DFD systems. The SMF integrates multi-scale feature fusion, ERC blocks, and other advanced components. It achieves a test accuracy of 97.79% with various near-ideal metrics and outperforms several existing studies in terms of classification performance. Robustness evaluation tests indicate near-ideal and consistent performance within the simulated scenario. However, the evaluation on a single dataset was observed to slightly limit generalizability, as shown in cross-dataset analysis. Future work will focus on the development of an end-to-end application for the proposed framework along with minimizing the generalizability issues. Examining how architectures can dynamically adapt to different computational limits can also be a promising future direction. In addition, integration of multimodal data in risk-sensitive domains with an emphasis on explainability can ensure transparent and trustworthy decision-making.

## Data Availability

Data will be made available on reasonable request to the corresponding author.

## Code availability

The source code used in this study is publicly available at: https://github.com/JayantiRout/SMF-Model-Deepfake-Image-Detection.git.
